# Non-Nucleosidic Analogues of Polyaminonucleosides and Their Influence on Thermodynamic Properties of Derived Oligonucleotides

**DOI:** 10.3390/molecules200712652

**Published:** 2015-07-13

**Authors:** Jolanta Brzezinska, Wojciech T. Markiewicz

**Affiliations:** Institute of Bioorganic Chemistry, Polish Academy of Sciences, Noskowskiego 12/14, 61-704 Poznań, Poland; E-Mail: jabrzoza@ibch.poznan.pl

**Keywords:** polyaminonucleoside analogue, non-nucleosidic analogue, spermine, putrescine, oligonucleotide, duplex DNA, thermodynamic stability

## Abstract

The rationale for the synthesis of cationic modified nucleosides is higher expected nuclease resistance and potentially better cellular uptake due to an overall reduced negative charge based on internal charge compensation. Due to the ideal distance between cationic groups, polyamines are perfect counterions for oligodeoxyribonucleotides. We have synthesized non-nucleosidic analogues built from units that carry different diol structures instead of sugar residues and functionalized with polyamines. The non-nucleosidic analogues were attached as internal or 5′-terminal modifications in oligodeoxyribonucleotide strands. The thermodynamic studies of these polyaminooligonucleotide analogues revealed stabilizing or destabilizing effects that depend on the linker or polyamine used.

## 1. Introduction

The polyanionic character of nucleic acids and their synthetic fragments is a barrier for their introduction into cells. Many laboratories try to improve nucleic acid delivery to cells by synthesis of cationic bioconjugates [1,2,3], bioconjugate analogues with reduced polyanionic character [4,5,6], or a great variety of polymeric transfection media [7,8,9,10,11,12]. On the other hand, there are studies to elucidate a specific mode of small cationic molecular drugs interactions with biomolecules (e.g., nucleic acids, proteins) useful in developing their more active analogues [13]. Among this clinically important class of compounds are antibiotics carrying aminosugar residues [14].

Chemically modified oligonucleotides have been utilized as indispensable materials for DNA gene therapy [15,16], gene regulation [17,18], chip technology [19,20] and recent nanotechnology [21,22,23] because of their hybridization affinity for target DNA and/or RNA molecules [24]. The polyanionic character of antisense and siRNA oligonucleotides is a major cause of insufficient cellular uptake and side effects such as binding to serum proteins. The combination of nucleotides with aminoalkyl chains greatly enhances the variety of possible structures as well as their potential application [24]. Thus, oligonucleotides possessing cationic functionalities in addition to the anionic phosphate backbone have been shown to exhibit promising properties [25,26,27,28]. Polyamines, putrescine, spermine and spermidine, are involved in the regulation of gene function [29,30]. *In vitro*, they stabilize DNA and RNA duplexes [31,32], especially ones with imperfect base pairing [33]. The distance between amino groups of three and four carbon atoms is practically the same as the distance between phosphate anions in the backbone of DNA making polyamines the perfect compounds for creating “zwitterionic” oligonucleotides [27]. It is known that polyamines interact with DNA and RNA in different ways. The electrostatic binding is performed via water molecules, by hydrogen bonding with polar functional groups or with hydrophobic surfaces of nucleobases. There have been numerous studies aimed at determining these interactions using NMR imaging, circular dichroism, Raman spectroscopy, IR spectroscopy, X-ray crystallography and differential scanning calorimetry. In spite of these extensive studies, precise mechanisms for the interaction between polyamine and DNA is still not fully understood *in vivo* [34].

**Figure 1:**
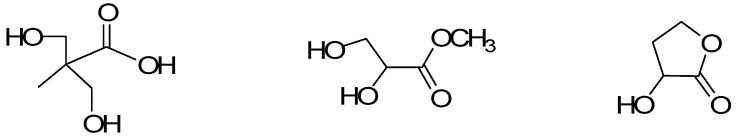
Substrates for the non-nucleosidic polyaminonucleoside analogues.

The non-nucleosidic analogs of polyaminonucleosides have not been examined so far. Therefore, our aim was to elaborate versatile procedures for synthesis of non-nucleosidic polyamine derivatives and learn their properties within oligonucleotide chains. The choice of carbon chain skeletons of analogues (Figure 1) was based on the extent of their commercial availability.

The obtained polyamine building blocks were incorporated at the 5′-end and internal positions within an oligodeoxyribonucleotide chain and the resulting conjugates were evaluated for their hybridization properties.

## 2. Results and Discussion

### 2.1. Chemistry

**Scheme 1:**
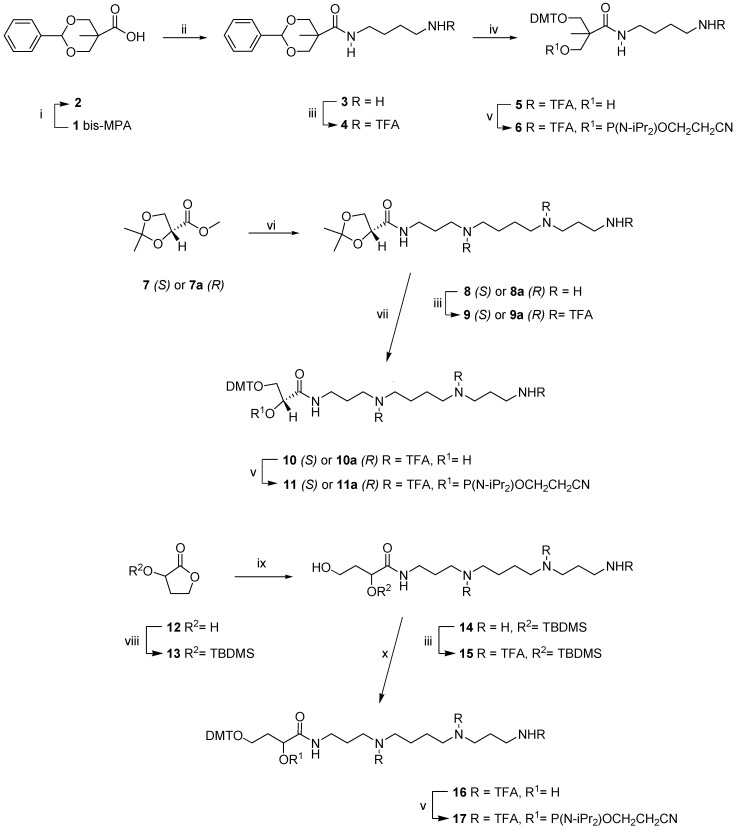
Synthetic routes to non-nucleosidic polyaminonucleoside analogues: (i) benzaldehyde, *p*-TsOH, DMF; (ii) SOCl_2_, putrescine, Et_3_N, DCM; (iii) (F_3_CCO)_2_O, pyridine; (iv) (a) 6 M HCl, reflux, (b) DMTCl, pyridine; (v) (iPr_2_N)_2_POCH_2_CH_2_CN, 5-ethylthio-1*H*-tetrazole, DCM; (vi) spermine, MeOH; (vii) (a) *p*-TsOH, MeOH, (b) DMTCl, pyridine; (viii) TBDMSCl, DMAP, imidazole, CH_3_CN; (ix) spermine, MW, 40 min; (x) (a) TEAHF, THF/dioxane, (b) DMTCl, pyridine.

The synthesis of the non-nucleosidic polyamine derived phosphoramidite building blocks containing different linkers is shown in Scheme 1.

We decided to use 2,2-bis(hydroxymethyl)-propionic acid (bis-MPA, **1**) that was earlier applied in dendrimer synthesis [35,36].

Bis-MPA (**1**) was protected with benzaldehyde [37] as it is more lipophilic than a isopropylidene group preferred to ease the final workup procedure and increase yield when using an excess of polar polyamines [38,39]. The protection of hydroxyl groups in bis-MPA gives rise to formation of two stereoisomers in the ratio ca 2:1, however, further reactions were carried out without separation of isomers. Several approaches for activation of carboxyl function (**2**) were checked. Reactions with 1,4-butylamine as a model amine in the presence of carbonyldiimidazole or 2-chloro-4,6-dimethoxy-1,3,5-triazine gave the expected n-butylamide derivative (results not shown). The best results were obtained when activation was performed with thionyl chloride in the presence of pyridine and traces of DMF. Thus, reaction with molar excess of putrescine (1,4-diaminobutane) at ambient conditions led to *N*-(4-aminobutyl)-5-methyl-2-phenyl-1,3-dioxane-5-carboxyamide **2** in 60% yield (Scheme 1).

We also synthesized the analogs based on the enantiomers (**7**) and (**7a**) which were coupled with excess of spermine in dry MeOH. Due to the lack of a chromophore in synthesized compounds, a reaction control was performed using TLC plates stained with a solution of fluorescein (free acid) in acetonitrile. Spermine derivatives (**8**, **8a**) were isolated by several extractions with large amounts of organic solvent and used in the next step without additional purification. The configuration of the branching point in these linkers is retained throughout the synthetic procedure. After protection of the amine function with trifluoroacetyl groups, 1,3-dioxolane was hydrolyzed to prepare polyamine non-nucleosidic derivatives for selective 4,4′-dimethoxytritylation. In order to avoid transacetylation of trifluoroacetyl groups during the isopropylidene cleavage of **9** we used *p*-TsOH instead acetic acid. Otherwise, amino groups would be at least in part protected with Ac instead of TFA and their deprotection with ammonia would not be possible under the conditions of the final deprotection of oligonucleotides. Commercially available precursor α-hydroxy-γ-butyrolactone (**12**) was used as a starting material to obtain a polyaminonucleoside analogue (**17**). This compound provides a three carbon distance between the phosphate groups after introduction into the oligodeoxyribonucleotide strand. It also contains a chiral carbon center with a secondary hydroxyl group and the carbonyl group corresponding to the 3′-hydroxyl and 2′-carbon of nucleosidic residues, respectively. The lactone-spermine conjugate was subsequently obtained after protection of the secondary hydroxyl group of the lactone (**13**) with lipophilic and a bulky t-butyldimethylsilyl (TBDMS) group. Thus, this eased the isolation of the product (**14**) resulting from the coupling reaction with spermine (Scheme 1). The reaction of protected lactone **13** and unprotected spermine was performed by microwave synthesis to achieve the desired product **14** with 64% yield, even when both reagents were used in equimolar ratio. To eliminate side reactions during the condensation step of DNA synthesis, the protection of putrescine and spermine amino functions with trifluoroacetyl groups was ensured (as previously described [40]. For all non-nucleosidic analogue yields of trifluoroacetylation ranged from 58% (spermine derivatives) up to 70% (putrescine derivative) (Scheme 1). Conversion to the corresponding phosphoramidite derivative was preceded by removal of protecting groups of hydroxyl function with HCl (**4**) and *p*-TsOH (**9**, **9a**) acids or Et_3_N*3HF (**15**). The protection 5′-OH-groups with a 4,4′-dimethoxytrityl group was used in a last step of the 3′-OH reaction with 2-cyanoethyl-*N*,*N*,*N′*,*N′*-tetraisopropylaminophosphane and 5-(ethylthio)-1*H*-tetrazole as the activator. However, due to similar physical properties, the separation of the products from impurities was difficult by silica gel column chromatography. This was resolved by precipitation from hexane. The spermine derivatives of *(R)* and *(S)* enantiomers of glyceric acid gained an additional chiral center as phosphoramidites (**11**, **11a**). In the case of the polyamine derivative of α-hydroxy-γ-butyrolactone, opening of the lactone ring **14** led to a mixture of enantiomers. Thus, after phosphitylation, compound **17** as a mixture of enantiomers was obtained. All amidites were lyophilized and were stable during long-term storage at −20 °C.

### 2.2. Oligonucleotides

**Figure 2:**
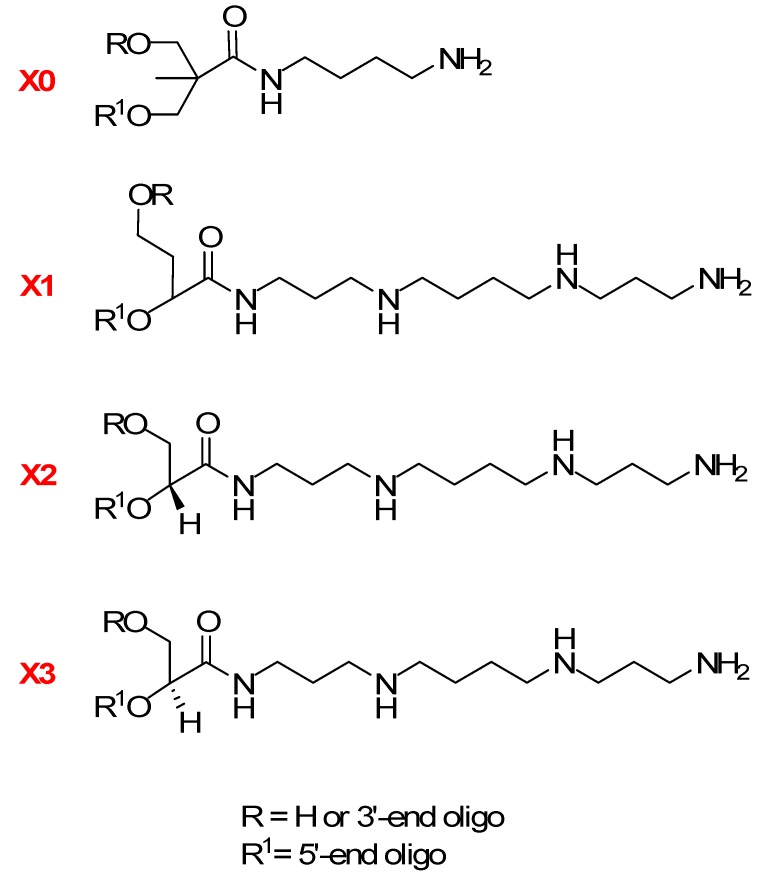
Non-nucleosidic polyamino-nucleoside analogues units in oligodeoxyribonucleotide strands.

The phosphoramidites (**6**, **11**, **11a**, **17**) were used for synthesis of polyamine analogs of oligodeoxyribonucleotides (Figure 2) using a 12-mer as a reference sequence (Table 1). The coupling efficiencies of these phosphoramidites were 53%–70% as determined by measuring detritylation. The modified oligodeoxyribonucleotides were obtained after a standard deprotection procedure, and their structures were confirmed by the MALDI-TOF (Table 1). The oligodeoxyribonucleotides **ON5**–**ON6** and **ON9** were used as pure enantiomers and **ON4** and **ON8** as mixtures of diastereoisomers.

**Table 1:** Sequences of oligodeoxyribonucleotides and MALDI-TOF MS data.

No.	Sequence 5′ → 3′ *^a^*	*m*/*z* [M − H]^−^	
		Calcd	Found
**ON1**	CTC AAG CAA GCT	3614.42	3613.08
**ON2**	CTC ACA TGC GCG	3606.40	3605.54
**ON3**	X0 CTC AAG CAA GCT	3994.42	3993.19
**ON4**	X1 CTC ACA TGC GCG	3973.12	3972.23
**ON5**	X2 CTC ACA TGC GCG	3959.54	3959.24
**ON6**	X3 CTC ACA TGC GCG	3959.54	3959.57
**ON7**	CTC AAG X0 CAA GCT	3994.42	3993.25
**ON8**	CTC ACA X1 TGC GCG	3973.12	3973.12
**ON9**	CTC ACA X2 TGC GCG	3959.54	3957.45

***^a^*** R = H for X0–X3 in ON3–ON6 and R = 3′-end oligo for X0–X2 in ON7–ON9.

### 2.3. Stability of Duplexes with Modified Oligodeoxyribonucleotides

**Figure 3:**
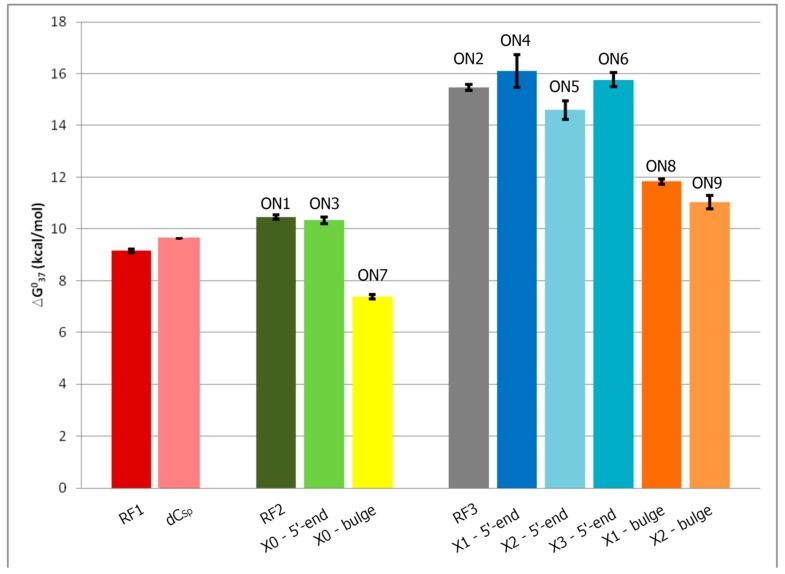
Changes in free energy (ΔG) of polyamine modified duplexes: RF-RF3- reference duplexes, dCSp—spermine modified (dC^Sp^ = 4-*N*-[4,9,13-triazatridecan-1-yl]-2′-deoxycytidine) duplex [34]; **ON3**, **ON7**—non-nucleosidic putrescine analogues (Table 2); **ON4–ON6** and **ON8–ON9**—non-nucleosidic spermine analogues (Table 2).

The influence of conjugated polyamines on the stability of the DNA duplexes was studied using UV melting spectra. It was shown previously that polyamine conjugation to oligodeoxyribonucleotides results in stabilizing of DNA duplexes and triplexes [2,3,4,38,39,41,42,43]. Recently, we described the NMR structure of a DNA duplex carrying a single spermine modified deoxycytidine unit [34]. This modification moderately stabilizes the DNA duplex (Figure 3, dC^Sp^ *vs.* RF1) and does not perturb the DNA structure.

The 5′-end dangling modifications in oligodeoxyribonucleotide duplexes usually increase duplex stability and do not show large sequence dependence [44,45]. Since the terminal unpaired nucleotides are not involved in base pairing, stacking, electrostatic, perhaps to some extent, hydrophobic interactions are responsible for the thermodynamic effects of the 5′- and 3′-dangling ends. The nearest-neighborhood model assumes the duplex region for a dangling end to have the same calculated thermodynamic stability as a matching blunt-end duplex [32,33].

Thus, the stability was increased when **X1** and **X3** (Scheme 1) were incorporated at the 5′ position (Table 1, entry **ON4** and **ON6**), but for incorporations of **X0** and **X2** (Scheme 1) a moderate destabilizing effect was observed (Table 1, entry **ON3** and **ON5**). Despite lack of a nucleobase and higher conformational flexibility at the 5′-end of the strand the observed stabilities are rather typical for 5′-end dangling units as described in the literature. Thermodynamic data (Table 2, Figure 1) show that **X3** results in ΔΔG (−0.3 kcal/mol) similar to unpaired nucleotide (−0.1 to −0.5 kcal/mol) at the 5'-end. The change of ΔG for **X1** is even higher (ΔΔG = −0.64 kcal/mol). These results suggest that the lack of a nucleobase does not affect the thermal stability of the duplexes. This might indicate that increased duplex stability is provided by the three positive charges in the spermine chain. In the case of the putrescine residue, **X0**, which introduces one positive charge only, a small decrease of duplex stability was observed. (Figure 1, **ON3**). One can conclude that the small decrease in duplex stability observed for **ON5** (Figure 1) containing spermine attached to the glycerol isomer (**X2**) is caused by the structure and configuration of this particular linker.

**Table 2:** DNA duplex stability of non-nucleosidic polyamine-modified oligodeoxyribonucleotides *^a^*.

Oligo	Average of Curve Fits				T_M_^−1^ *vs.* log (C_T_/4) Plots					
	−ΔH° [kcal/mol]	−ΔS° [eu]	−ΔG°_37_ [kcal/mol]	T_M_ [°C]	−ΔH° [kcal/mol]	−ΔS° [eu]	−ΔG°_37_ [kcal/mol]	T_M_ [°C]	ΔΔG°_37_ [kcal/mol]	ΔT_M_ *^b^* [°C]
**A. Thermodynamic parameters of references duplexes**										
**ON1 *^c^***	67.8 ± 9.0	185.6 ± 27.8	10.27 ± 0.44	55.1	72.6 ± 2.0	200.6 ± 6.2	10.45 ± 0.08	54.7	­	­
**ON2 *^d^***	115.9 ± 4.8	322.1 ± 14.2	16.00 ± 041	64.6	108.5 ± 1.8	300.1 ± 5.4	15.46 ± 0.12	64.8	­	­
**B. Thermodynamic parameters of duplexes with the polyamine analog as a dangling end**										
**ON3 *^c^***	63.3 ± 6.8	171.5 ± 20.9	10.12 ± 0.37	55.7	69.09 ± 3.1	189.4 ± 9.7	10.33 ± 0.13	55.0	+0.12	−0.3
**ON4 *^d^***	131.8 ± 10.4	368.8 ± 31.0	17.47 ± 0.80	65.1	114.0 ± 8.3	315.9 ± 24.9	16.10 ± 0.64	65.4	−0.64	+0.6
**ON5 *^d^***	97.8 ± 9.4	268.4 ± 28.1	14.57 ± 0.68	64.8	98.9 ± 5.1	271.9 ± 15.4	14.59 ± 0.36	64.5	+0.87	−0.3
**ON6 *^d^***	106.1 ± 4.7	292.4 ± 14.0	15.43 ± .0.37	65.4	110.9 ± 3.9	307.0 ± 11.7	15.76 ± 0.27	65.1	−0.3	+0.3
**C. Thermodynamic parameters of duplexes with the polyamine analog as a bulge**										
**ON7 *^c^***	62.7 ± 16.7	178.2 ± 53.8	7.50 ± 0.20	41.9	59.0 ± 4.2	166.5 ± 13.7	7.38 ± 0.08	41.5	+3.07	−13.2
**ON8 *^d^***	71.6 ± 6.0	194.4 ± 18.3	11.31 ± 0.35	59.2	82.0 ± 2..0	226.3 ± 6.2	11.83 ± 0.10	58.4	+3.63	−6.4
**ON9 *^d^***	81.2 ± 11.5	225.0 ± 35.6	11.45 ± 0.48	57.0	70 ± 6.4	192.1 ± 20.0	11.03 ± 0.25	58.1	+4.43	−6.7

*^a^* Solutions are 100 mM NaCl, 20 mM sodium cacodylate, 0.5 mM Na_2_EDTA, pH 7; *^b^* Calculated for 10^−4^ M total strand concentration; *^c^* Complementary strand 5′-AGC TTG CTT GAG-3′; *^d^* Complementary strand 5′-CGC GCA TGT GAG-3′.

Next, we investigated the influence of polyamine derivatives **X0**, **X1** and **X2** on duplex stability, inserted as bulges in the middle of the sequence (Table 1, entry **ON7**–**ON9**). The changes of duplex stability caused by single nucleotide bulges differ and depend on the type of flanking bases [46,47]. Incorporation of **X0**–**X2** resulted in a lowering of melting temperature independently of polyamine residue. However, the melting data suggest that the duplex formation occurs with the proper W-C base pairing despite of a slight increase in free energy for **ON7**–**ON9** (Table 2, ΔΔG *ca.* 3–4.5 kcal/mol). Moreover, the effect in **ON7** where the putrescine bulge (**X0**) is flanked by GC/CG pairs is practically the same as for **ON8** and **ON9** (spermine bulge, **X1** and **X2**) flanked by AT/TA pairs. This can be attributed to the stronger stabilizing effect of a spermine residue when compared to putrescine—three positive charges *vs.* one. Yet, even three positive charges of a spermine residue do not neutralize the bulge effect as such. The observed changes of ΔG for the studied duplexes (**ON7**–**ON9**) are in the range observed for duplexes with a single bulge (ΔΔG, 2–6 kcal/mol) [46,47].

Linkers X1 and X2 differ in length by one carbon and this additional carbon in X1 makes this linker somehow less rigid, allowing for more favorable placement of the polyamine residue. Thus, the energy gain of 1.5 kcal/mol in **ON4** when compared to **ON5**. Therefore, **ON4** duplex is more stable than the unmodified duplex. When the same modified linkers (X1 and X2) are inserted as bulges in a more spatially “demanding environment”, the difference of free energies **ΔΔ**G°_37_ of **ON8** (+3.63) and **ON9** (+4.43) is smaller (0.8 kcal/mol). The influence of linker structure is less profound when the modification is inserted in the middle of chain.

Linkers X2 and X3 carrying a spermine residue differ only in the configuration of carbon (*S* and *R* respectively). Comparison of **ΔΔ**G°_37_ of ON6 (−0.3 kcal/mol) and ON5 (+0.87 kcal/mol) suggests that X3 allows more favorable placement of polyamine residue This seems to indicate that in this case the spermine residue attached to a glycerol linker with *R*‑configuration (X3) is closer to the duplex charged surface.

We would like to conclude that the overall effect of DNA modification with polyamine analogues seems to offer a convenient way to modify nucleic acids properties. An attachment of polyamine residues via various open chain linkers allows to maintain the general scheme of base pairing. Moreover, the structure and configuration of the linkers seems to have a higher influence on stability when placed at the 5′-end of DNA duplexes.

## 3. Experimental Section 

### 3.1. General Methods

All reagents were of analytical grade, obtained from commercial resources and used without further purification. For synthesis, solvents with quality pro analysis were used. Solvents were dried and distilled following standard methods and kept over molecular sieve. All reactions were carried at room temperature unless described otherwise. Column chromatography was performed with silica gel (Merck KGaA, Darmstadt, Germany, 200–630 mesh) and TLC was carried out on precoated plates (Merck silica gel 60, F_254_). All NMR spectra were recorded at 298 K on Bruker AVANCE II (400 MHz, ^1^H, ^13^C) and Varian Unity (300 MHz, ^31^P) spectrometers (Bruker BioSpin GmbH, Rheinstetten, Germany and Varian, Inc., Palo Alto, CA, USA). Chemical shifts (δ) are reported in parts per million (ppm). *J* values are given in Hz. Mass spectra were recorded on the MicroTofQ mass spectrometer with electrospray ionization (ESI) sources (ESI source voltage of 3.2 kV, nebulization with nitrogen at 0.4 bar, dry gas flow of 4.0 L/min at temperature 220 °C) and Bruker Autoflex MALDI-TOF (Bruker Daltonik GmbH, Bremen, Germany). Microwave reactions was performed in domestic microwave oven (800 W, Amica, Wronki, Poland). Some NMR (Figures S1–S16) and MS (Figures S17–S22) spectra are available in Supplementary.

### 3.2. Synthesis of Monomers

*Benzylidene-2,2-bis(oxymethyl)propionic acid* (**2**) [37]. Benzaldehyde (4.2 mL, 40.7 mmol) was added to a well-stirred solution of 2,2-bis(hydroxymethyl)-propionic acid (**1**) (5 g, 37 mmol) in DMF (30 mL), followed by catalytic *p*-TsOH (0.355 g, 1.85 mmol) with stirring at room temperature for 4 days. The reaction was quenched with NH_4_OH/EtOH (1 mL, 1:1). The solvent was evaporated and the residue dissolved in DCM (100 mL) and washed with NaHCO_3_ (2 × 100 mL). Organic extracts were dried over anhydrous MgSO_4_ and filtered then evaporated under reduced pressure. The residue was purified by recrystallization from DCM to obtain product **2** as mixture of two isomers (77%). ^1^H-NMR (CDCl_3_); isomer A: 7.28–7.46 (m, 5H, Ph), 5.45 (s, 1H, PhCHO_2_), 4.65 (d, 2H, *J =* 11.7 Hz, OCH_2_CCO, 3.67 (d, 2H, *J =* 11.7 Hz, OCH_2_CCO), 1.02 (s, 3H, CH_3_); isomer B: 7.28–7.51 (m, 5H, Ph), 5.41 (s, 1H, Ph*CH*O_2_), 4.14–4.06 (q, 4H, *J =* 11.23 Hz, *J =* 9.27 Hz, 2CH_2_), 1.57 (s, 3H, CH_3_). ^13^C-NMR (CDCl_3_): 178.89 (C=O), 137.58 (C, Ph), 129.08 (CH, Ph), 128.23 (CH, Ph), 128.23 (CH, Ph), 101.86 (CH), 73.43 (CH_2_), 42.17 (CMe), 17.78 (CH_3_). MS (ESI) *m*/*z*: calcd for C_12_H_14_O_4_ 221.0819 [M − H]^−^, found 221.182.

*N-(4-Aminobutyl)-5-methyl-2-phenyl-1,3-dioxane-5-carboxamide* (**3**). Thionyl chloride (1.26 mL, 17.8 mmol) was added dropwise to ice-cooled solution of **2** (2 g, 8.9 mmol) in 45 mL DCM containing 10% of pyridine and 3 drops of DMF. The mixture was stirred for 2 h at room temperature, and the excess thionyl chloride was removed by several co-evaporations with a mixture of DCM and toluene. The brown residue was dissolved in DCM (46 mL) and the temperature was lowered to −10 °C. Putrescine (4.47 mL, 44.5 mmol) and triethylamine (1.4 mL) in DCM (2 mL) was added after 15 min. The mixture was stirred for 1h at room temperature, and the reaction was quenched with saturated aqueous NaHCO_3_ (3 mL). The mixture was extracted with DCM (2 × 100 mL). The combined organic extracts were dried over anhydrous MgSO_4_ and concentrated under vacuum and purified by chromatography with 4% MeOH in DCM as the eluent to give **3** (0.910 g, 60% yield) as a yellow oil. ^1^H-NMR (CDCl_3_): 7.34–7.43 (m, 5H, Ph), 7.03 (t, 1H, NHCO), 5.48 (s, 1H, Ph*CH*O_2_), 4.33 (d, 2H, *J =* 12 Hz, OCH_2_CCO), 3.78 (d, 2H, *J =* 12 Hz, OCH_2_CCO), 3.33 (q, 2H, *J =* 6.3 Hz, *J =* 5.8 Hz, CONH*CH_2_*), 3.21 (q, 2H, *J =* 6.3 Hz, *J =* 5.8 Hz, NH_2_*CH_2_*, putrescine), 1.5–1.56 (m, 4H, 2 × CH_2_, putrescine), 1.04 (s, 3H, CH_3_); ^13^C-NMR (CDCl_3_): δ (ppm) 178.89 (C=O), 137.45 (C, Ph), 128.7 (CH, Ph), 128.03 (CH, Ph), 101.22 (CH), 75.07 (CH_2_), 47.6 (CMe), 47.6 (CH_2_, putrescine), 26.53 (CH_2_, putrescine), 18.03 (CH_3_); MS (ESI) *m*/*z*: calcd for C_16_H_24_N_2_O_3_ 293.186 [M + H]^+^, found 293.2037.

*5-Methyl-2-phenyl-N-(4-(2,2,2-trifluoroacetamid)butyl)-1,3-dioxane-5-carboxamide* (**4**) **3** (0.430 g, 1.47 mmol) was co-evaporated with pyridine (3 × 5 mL), dissolved in pyridine (15 mL), followed by the addition of N-methylimidazole (0.16 mL, 1.46 mmol). Then, trifluoroacetic anhydride (0.593 mL, 4.41 mmol) was added dropwise to the mixture cooled at 0 °C. The mixture was stirred for 30 min and then poured into saturated aqueous NaHCO_3_ (30 mL), and extracted with DCM (3 × 50 mL). The combined organic extracts were dried over anhydrous Na_2_SO_4_ and concentrated under vacuum. The residue was purified by chromatography using DCM as the eluent to give **4** (0.500 g, 52% yield) as an oil. ^1^H-NMR (CDCl_3_): 7.33–7.45 (m, 5H, Ph), 7.03 (t, 1H, NHCO), 5.5 (s, 1H, PhCHO_2_), 4.38 (d, 2H, *J =* 11.7 Hz, OCH_2_CCO), 3.78 (d, 2H, *J =* 11.7 Hz, OCH_2_CCO), 3.2–3.4 (m, 4H, 2NH*CH_2_*), 1.49–1.54 (m, 4H, CH_2_, putrescine), 1.06 (s, 3H, CH_3_). ^13^C-NMR (CDCl_3_): 182.83 (C=O), 158.9 (C=O), 137.45 (C, Ph), 128.7 (CH, Ph), 128.03 (CH, Ph), 125.53 (C, CF_3_), 101.22 (CH), 75.07 (CH_2_), 47.4 (CMe), 45.3 (CH_2_, putrescine), 26.53 (CH_2_, putrescine), 18.03 (CH_3_); ^19^F NMR 1.78 (s, 3F, CF_3_); MS (ESI) *m*/*z*: calcd for C_18_H_23_F_3_N_2_O_4_ 389.1683 [M + H]^+^, found 389.1627. 

*3-(4,4′-Dimethoxytrityl)-2-(hydroxymethyl)-2-methyl-N-(4-(2,2,2-trifluoroacetamido)butyl)propanamide* (**5**). **4** (0.240 g, 0.61 mmol) was dissolved in a mixture conc. aq HCl/EtOH (1:5, *v*/*v*) and refluxed for 72 h. The excess of HCl was removed by several co-evaporations with a mixture of methanol and toluene and finally a brown oil with was dried by co-evaporation with anhydrous pyridine (2 × 5 mL) and dissolved in anhydrous pyridine (2.4 mL). To this solution 4,4′-dimethoxytrityl chloride (0.243 g, 0.72 mmol) was added and the reaction was quenched after 3 h by adding saturated aqueous NaHCO_3_. The resulting solution was extracted with DCM and the combined organic extracts were washed with brine, dried over Na_2_SO_4_ and concentrated under vacuum. The residual yellow oil was purified by chromatography with 5% MeOH in DCM as the eluent to give 0.18 g (49%) **5** as a white foam. ^1^H-NMR (CDCl_3_): δ (ppm) 7.42–7.2 (m, 10H, Ph, NHCO), 7.04 (t, 1H, NHCO), 6.89 (m, 4H, Ph), 3.82 (s, 3H, OCH_3_), 3.83–3.62 (m, 4H, OCH_2_CCO), 3.43–3.23 (m, 4H, 2NH*CH_2_*), 1.4–1.8 (m, 4H, 2CH_2_, putrescine), 1.2 (s, 3H, CH_3_). ^13^C-NMR (CDCl_3_): δ (ppm) 174.4 (C=O), 158.5 (2C, *C*OCH_3_), 158.4 (C=O, TFA), 144.6 (C, Ph), 135.5 (C, Ph), 130.0 (CH, Ph), 128.3 (CH, Ph), 128.0 (CH, Ph), 127.8 (CH, Ph), 126.8 (C, CF_3_), 113.1 (CH, Ph), 86.2 (C), 66.9 (CH_2_), 65.5 (CH_2_), 55.1 (CH_3_), 47.5 (C, CMe), 43.2 (CH_2_, putrescine), 24.5 (CH_2_, putrescine), 18.7 (CH_3_); MS (ESI) *m/z*: calcd for C_32_H_37_F_3_N_2_O_6_ 603.2676 [M + H]^+^, found 603.261.

*3-[(4,4′-Dimethoxytrityl)-2-(hydroxymethyl)-2-methyl-N-(4-(2,2,2-trifluoroacetamido)butyl)propanamide]phosphoramidite* (**6**). 2-Cyanoethyl-*N*,*N*,*N*,*N*-tetraisopropylphosphoramidite (0.9 mL, 0.3 mmol) was added to the solution of **5** (0.143 g, 0.234 mmol) and 5-(ethylthio)-1*H*-tetrazole (0.0273 g, 0.21 mmol) in dichloromethane (1.2 mL) and the mixture was stirred at room temperature. After 2 h, TLC revealed complete reaction. The mixture was diluted with dichloromethane, washed with saturated sodium bicarbonate solution and the organic extracts were dried over Na_2_SO_4_. The product was purified by silica gel chromatography with benzene–Et_3_N (10%) and lyophilisation from benzene to give **6** (134 mg, 70% yield) as a white powder. ^31^P-NMR (C_6_H_6_): δ (ppm) 148.30; 148.54. MS (ESI) *m*/*z*: calcd for, C_41_H_54_F_3_N_4_O_7_P [M + H]^+^ 803.3755, found 803.371. 

*(S)-N-(4,9,13-Triazatridecan-1-yl)-2,2-dimethyl-1,3-dioxolane-4-carboxyamide* (**8**). The methyl (*S*)-2,2-dimethyl-1,3-dioxalane-4-carboxylate (**7**) (0.45 mL, 3 mmol), spermine (2.4 g, 12 mmol) were dissolved in anhydrous methanol (1 mL) and the resulting solution was stirred at room temperature for 48–72 h at 25–35 °C. The solvent was evaporated under reduced pressure and the residue was purified by column chromatography over silica gel (MeOH/MeNH_2_/H_2_O) to give **8** (720 mg, 70% yield) as yellow oil. ^1^H-NMR (CDCl_3_): δ (ppm) 7.22 (m, 1H, NHCO), 4.45 (q, 1H, *J =* 5.37 Hz, *J =* 2.44 Hz, COCH), 4.25 (t, 1H, *J =*7.8, OCHCO), 4.05 (q, 1H, *J =* 5.37 Hz, *J =* 3.41 Hz, COCH), 3.38–3.24 (m, 2H, CONH*CH_2_*), 2.53–2.65 (m, 6H, CH_2_), 2.05 (2H, NH), 1.66 (m, 2H, spermine), 1.49 (m, 2H, spermine), 1.44 (s, 3H, CH_3_), 1.44 (s, 3H, CH_3_); ^13^C-NMR (CDCl_3_): δ (ppm) 171.3 (C=O), 110.6 (OCO), 74.8 (C), 67.6 (C), 49.1 (CH_2_), 46.8 (CH_2_), 37.1 (CH_2_), 28.6 (CH_2_), 27.1 (CH_2_), 26.0 (CH_2_), 24.8 (CH_3_). MS (ESI) *m*/*z*: calcd for C_16_H_34_N_4_O_3_ [M + K]^+^ 369.2262, found 370.2801. 

*(R)-N-(4,9,13-Triazatridecan-1-yl)-2,2-dimethyl-1,3-dioxolane-4-carboxyamide* (**8a**). The methyl (*R*)-2,2-dimethyl-1,3-dioxalane-4-carboxylate (**7a**) (0.45 mL, 3 mmol) was converted into compound **8a** following the above procedure (566 mg, 55% yield). ^1^H-NMR (CDCl_3_): δ (ppm) 7.19 (m, 1H, NHCO), 4.45 (q, 1H, *J =* 5.37 Hz, *J =* 2.44 Hz, COCH), 4.26 (t, 1H, *J* = 7.8 Hz, OCHCO), 4.05 (q, 1H, *J =* 5.37 Hz, *J* *=* 3.41 Hz, COCH), 3.43–3.3 (m, 2H, CONH*CH*_2_), 2.74–2.59 (m, 6H, CH_2_), 1.76 (m, 2H, spermine), 1.63 (m, 2H, spermine), 1.47 (s, 3H, CH_3_), 1.37 (s, 3H, CH_3_); ^13^C-NMR (CDCl_3_): δ (ppm) 171.3 (C=O), 110.6 (OCO), 74.8 (C), 67.6 (C), 49.1 (CH_2_), 46.8 (CH_2_), 37.1 (CH_2_), 28.6 (CH_2_), 27.1 (CH_2_), 26.0 (CH_2_), 24.8 (CH_3_). MS (ESI) *m*/*z*: calcd for C_16_H_34_N_4_O_3_ [M + H]^+^ 331.2704, found 331.2701.

*(S)-N-[Tris(2,2,2-trifluoroacet-1-yl)-4,9,13-triazatridecane]-2,2-dimethyl-1,3-dioxolane-4-carboxyamide* (**9**). **8** (0.200 g, 0.6 mmol) was co-evaporated with anhydrous pyridine (3 × 5 mL) and redissolved in pyridine (6 mL). Trifluoroacetic anhydride (0.5 mL, 3.6 mmol) was added dropwise to a cooled and stirred solution at 0 °C. The solution was warmed to room temperature and stirred for 30 min, quenched with NaHCO_3_ and extracted with CH_2_Cl_2_. The combined organic extracts were washed with saturated aqueous NaCl, dried over Na_2_SO_4_ and concentrated under vacuum. The residual oil was purified by chromatography with 2% MeOH in DCM as the eluent to give **9** (0.2 g, 54% yield) as a white foam. ^1^H-NMR (CDCl_3_): δ (ppm) 7.09 (m, 1H, NHCO), 6.72 (m, 1H, NHCO), 4.46 (q, 1H, *J =* 5.34 Hz, *J =* 3.78 Hz, COCH), 4.07 (q, 1H, *J =* 5.34 Hz, *J =* 3.78 Hz, COCH), 4.28 (t, 1H, *J =* 7.64 Hz, OCHCO), 3.49–3.25 (m, 8H, NH*CH_2_*CH_2_, spermine), 1.86–1.78 (m, 2H, spermine), 1.61 (m, 2H, spermine), 1.49 (s, 3H, CH_3_), 1.39 (s, 3H, CH_3_). ^19^F-NMR: 6.98–7.22 (m, 9F, 3CF_3_). MS (ESI) *m*/*z*: calcd for C_22_H_30_F_9_N_4_O_6_ [M − H]^−^ 617.2022, found 617.3021.

*(R)-N-[Tris(2,2,2-trifluoroacet-1-yl)-4,9,13-triazatridecane]-2,2-dimethyl-1,3-dioxolane-4-carboxyamide* (**9a**). **8a** (0.350 g, 1.05 mmol) was converted into **9a** (0.368 g, 58% yield) following procedure for **9**. ^1^H-NMR (CDCl_3_): δ (ppm) 7.17 (m, 1H, NHCO), 6.76 (m, 1H, NHCO), 4.47 (q, 1H, *J* = 7.2 Hz, *J =* 3.3 Hz, COCH), 4.07 (m, 1H, COCH), 4.29 (t, 1H, *J* = 8 Hz, OCHCO), 3.48–3.26 (m, 8H, NH*CH_2_*CH_2_, spermine), 1.88–1.82 (m, 2H, spermine), 1.62 (m, 2H, spermine), 1.49 (s, 3H, CH_3_), 1.39 (s, 3H, CH_3_). ^19^F-NMR: 6.98–7.22 (m, 9F, 3CF_3_). MS (ESI) *m*/*z* calcd for C_22_H_31_F_9_N_4_O_3_ [M + K]^+^ 657.1737, found 657.175.

*(S)-N-[Tris(2,2,2-trifluoroacet-1-yl)-4,9,13-triazatridecane]-3-(4,4′-dimethoxytrityl)-3-hydroxypropanamide* (**10**). To a solution of **9** (0.500 g, 0.75 mmol) in MeOH (4 mL), *p*-toluenosulfonic acid (0.03 g, 0.15 mmol) was added at room temperature. The mixture was stirred for ca 30 h at room temperature, and solvent was evaporated. The mixture was diluted with ethyl acetate and washed with aq. saturated NaHCO_3_. The combined organic extracts were dried over Na_2_SO_4_ and concentrated under vacuum. The resulting crude was co-evaporated with pyridine (4 × 5 mL) and dissolved in pyridine (2.5 mL). 4,4′-Dimethoxytrityl chloride (0.330 g, 0.975 mmol) was added in portions at room temperature. The mixture was stirred for 3 h at room temperature, and the reaction was quenched with methanol (1 mL) and saturated aq. NaHCO_3_ (10 mL). The mixture was extracted with DCM. The combined organic extracts were dried over MgSO_4_ and concentrated under vacuum. The resulting orange oil was purified by chromatography with 2%–3% MeOH in CH_2_Cl_2_ (with 0.2% of pyridine by vol.) as the eluent to give **10** (0.494 g, 69% yield) as a white solid. ^1^H-NMR (CDCl_3_): δ (ppm) 7.39–6.8 (m, 15H, DMT, NHCO), 4.19–4.13 (m, 1H, OCHCO), 3.75 (s, 6H, 2OCH_3_), 3.51–3.22 (m, 14H), 1.99–175 (m, 4H, spermine), 1.68–1.55 (m, 4H, spermine). ^13^C-NMR (CDCl_3_): δ (ppm) 171.9 (C), 171.7 (C), 158.7 (C), 144.4 (C), 135 (C), 129.9 (CH), 127,9 (CH), 127 (CH), 125.8 (C), 121 (C), 113.2 (CH), 86.8 (C), 77.2 (CH), 67.7 (CH_2_), 55.2 (CH_3_), 46.3 (CH_2_), 36.1 (CH_2_), 35.1 (CH_2_), 29.7 (CH_2_), 27.1 (CH_2_). MS (ESI) *m*/*z*: calcd for C_40_H_45_F_9_N_4_O_8_ [M + Na]^+^ 903.2991, found 903.2938; calcd for C_40_H_45_F_9_N_4_O_8_ [M + K]^+^ 919.2731, found 919.266. 

*(R)-N-[Tris(2,2,2-trifluoroacet-1-yl)-4,9,13-triazatridecane]-3-(4,4′-dimethoxytrityl)-3-hydroxypropanamide* (**10a**). **9a** (0.350 g, 1.05 mmol) was converted into **10a** (0.383 g, 54% yield) following procedure for **10**. ^1^H-NMR (CDCl_3_): δ (ppm) 7.39–6.8 (m, 15H, DMT, NHCO), 4.2–4.12 (m, 1H, OCHCO), 3.75 (s, 6H, 2OCH_3_), 3.48–3.18 (m, 14H), 1.91–1.74 (m, 4H, spermine), 1.63–1.51 (m, 4H, spermine). MS (ESI) *m*/*z*: calcd for C_40_H_45_F_9_N_4_O_8_ [M − H]^−^ 879.3015, found 879.3028. 

2-(S)-[(4,4′-Dimethoxytrityl)-3-(hydroxymethyl)-N-((2,2,2-trifluoroacet-1-yl)-4,9,13-triazatridecane)propanamide]phosphoramidite (**11**). The compound **10** was converted into compound **11** following procedure for **6**. Except purification: crude phosphoramidite was trice precipitated from hexane and finally lyophilized from benzene to give **11** (0.210 g, 52% yield) as a white light solid. ^31^P-NMR (C_6_H_6_): δ (ppm) 151.89; 151.68; 148.99; 148.68; 148.56; 146.81. MS (ESI) m/z: calcd for C_49_H_62_F_9_N_6_NaO_9_P [M + Na]^+^ 1103.4070, found 1103.4575; calcd for C_49_H_62_F_9_KN_6_O_9_P [M + K]^+^ found 1119.4509.

2-(R)-[(4,4′-Dimethoxytrityl)-3-(hydroxymethyl)-N-((2,2,2-trifluoroacet-1-yl)-4,9,13-triazatridecane)propanamide]phosphoramidite (**11a**). **10a** (0.200 g, 0.226 mmol) was converted into compound **11a** (0.128 g, 52% yield) according to procedure for **11**. ^1^H-NMR (CDCl_3_): δ (ppm) 7.14–7.38 (m, 9H, DMT), 6.78 (m, 4H, DMT), 4.8 (m, 1H, COCH), 4.65 (m, 1H, OCHCO), 3.73 (s, 6H, 2OCH_3_), 3.12–3.55 (m, 16H, CH_2_OH, spermine, CH_2_OP) 2.4–2.78 (m, 5H, 2CH, iPr, CH_2_CN), 1.41–1.92 (m, 8H, spermine) 1.17–1.23 (m, 12H, iPr). ^31^P-NMR (C_6_H_6_): δ (ppm) 151.76; 151.42; 149.24; 149.05; 148.96; 148.83. MS (ESI) m/z: calcd for C_49_H_62_F_9_N_6_O_9_P [M − H]^−^ 1079.4094, found 1079.63. 

*3-(tert-Butyldimethylsilyloxy)butyrolactone* (**13**) [48]. *tert*-Butyldimethylchlorosilane (1.50 g, 9.63 mmol), imidazole (0.470 g, 3.21 mmol) and *N*,*N*-dimethylaminopyridine (0.392 g, 3.21 mmol) were added to a solution of α-hydroxy-γ-butyrolactone (**12**) (0.25 mL, 3.21 mmol) in anhydrous CH_3_CN (16 mL). The reaction mixture was stirred at room temperature for 16 h. Then, solvent was evaporated under reduced pressure. The residue was partitioned between saturated aq. NaHCO_3_ and Et_2_O (3 × 50 mL). The organic extracts were washed with brine, dried over MgSO_4_. The crude residue was purified by column chromatography (eluent hexane/Et_2_O 4:1→2:1) to give compound **12** as colourless oil (0.485 g, 70% yield). ^1^H-NMR (CDCl_3_): δ (ppm) 4.35–4.4 (m, 2H, CH_2_O), 4.15–4.2 (dt, 1H, OCH), 2.16–2.5 (m, 1H, O*CH*CH_2_), 0.9 (s, 9H, 3CH_3_, t-Bu), 0.16 (s, 3H, CH_3_CSi), 0.14 (s, 3H, CH_3_CSi); ^13^C-NMR (CDCl_3_): δ (ppm) 175.86 (C=O), 68.19 (CH), 64.72 (CH_2_), 33.3 (CH_2_), 25.61 (CH_3_), 18.19 (SiC(CH_3_)_3_), 5.5 (CH_3_); MS (ESI) *m/z*: calcd for C_10_H_20_O_3_Si [M − H]^−^ 215.1103, found 215.0295.

*N-(4,9,13-Triazatridecan-1-yl)-2-(tert-butylodimethylsiloxy)-butyramide* (**14**). A solution of **13** (0.216 g, 1 mmol) and spermine (0.202 g, 1 mmol) in a small (10 mL) Erlenmeyer flask was kept in microwave oven for 45 min. The flask was cooled down and the mixture was diluted with DCM (20 mL), washed with NaHCO_3_ (3 × 30 mL), brine (30 mL), and organic layers dried over MgSO_4_. After evaporation, the residue was chromatographed using MeOH/H_2_O/MeNH_2_ as the eluent to give **14** (0.266 g, 64% yield) as oil. ^1^H-NMR (CDCl_3_): δ (ppm) 7.02 (t, 1H, NHCO), 4.25 (t, 1H, *J* = 6.01 Hz, COCHOSi), 3.71 (q, 2H, *J =* 5.89 *CH_2_*OH), 3.38–3.32 (m, 2H, spermine), 2.65–2.58 (m, 4H), 1.99–1.94 (m, 2H, OCH*CH*_2_), 1.76 (m, 2H, spermine), 1.63 (m, 2H, spermine), 0.91 (s, 9H, 3CH_3_, t-Bu), 0.11 (s, 3H, CH_3_CSi), 0.08 (s, 3H, CH_3_CSi). MS (ESI) *m*/*z*: calcd for C_20_H_47_N_4_O_3_Si [M + H]^+^ 419.3417, found 419.3539. 

*N-[Tris(2,2,2-trifluoroacet-1-yl)-4,9,13-triazatridecane]-2-(tert-butyldimethylsiloxy)-butyramide* (**15**). **14** (0.266 g, 0.64 mmol) was co-evaporated with anhydrous pyridine (3 × 5 mL) and redissolved in pyridine (6 mL). Trifluoroacetic anhydride (0.517 mL, 3.83 mmol) was added dropwise at 0 °C. The solution was warmed to room temperature and stirred for 30 min., quenched with NaHCO_3_ and extracted with DCM (3 × 30mL). The combined organic extracts were washed with brine, dried over MgSO_4_ and purified by chromatography with 2%–3% MeOH in DCM as the eluent to give **15** (0.297 g, 66% yield) as an oil. ^1^H-NMR (CDCl_3_): δ (ppm) 7.09 (bs, 1H, NHCO), 6.83 (bs, 1H, NHCO), 4.27 (t, 1H, COCHOSi), 3.73 (q, 2H, *J =* 5.89 Hz, *CH_2_*OH), 3.47–3.15 (m, 6H), 2.01–1.75 (m, 4H), 1.63 (m, 2H, spermine), 0.93 (s, 9H, 3CH_3_, t-Bu), 0.12 (s, 3H, CH_3_CSi), 0.10 (s, 3H, CH_3_CSi). ^19^F-NMR 6.98–7.22 (m, 9F, CF_3_). MS (ESI) *m*/*z*: calcd for C_26_H_43_F_9_N_4_O_6_Si [M − H]^−^ 705.2730, found 705.2809. 

*N-[Tris(2,2,2-trifluoroacet-1-yl)-4,9,13-triazatridecane]-4-(4,4′-dimethoxytrityl)-butyramide* (**16**). TEAHF [49,50] solution (1 M in THF/dioxane, 1.08 mmol, 1 mL) was added at room temperature to a solution of **15** (0.254 g, 0.36 mmol) in THF (1 mL) and kept overnight at room temperature. The reaction was quenched with NH_4_Cl solution and extracted with ethyl acetate (3 × 15 mL). The combined extracts were dried over MgSO_4_, concentrated under vacuum. The residue was co-evaporated with anhydrous pyridine (4 × 5 mL) and redissolved in pyridine (2 mL). 4,4′-Dimethoxytrityl chloride (1.2 eq) was added in portions at room temperature. The mixture was stirred for 3 h at room temperature, and the reaction was quenched with methanol (1 mL) and saturated aqueous NaHCO_3_ (10 mL). The mixture was extracted with DCM. The combined organic extracts were dried over MgSO_4_, concentrated under vacuum and the resulting orange oil was purified by chromatography with 2%–3% MeOH in DCM (with 0.2% of pyridine) as the eluent to give **16** (0.231 g, 72% yield ) as a white solid. ^1^H-NMR (CDCl_3_): δ (ppm) 7.19–7.36 (m, 9H, DMT), 6.78 (d, 4H, *J =* 8.6 Hz, DMT), 4.21 (dt, 1H, CO*CH*OH), 3.74 (s, 6H, 2OCH_3_), 3.0–3.44 (m, 14H, CH_2_OH, spermine), 2.18–2.19 (m, 2H, OCH*CH_2_*), 1.56–1.88 (m, 8H, spermine). MS (ESI) *m*/*z*: calcd for C_40_H_46_F_9_N_4_O_8_ [M + K]^+^ 933.2887, found 933.279.

*2-(4,4′-Dimethoxytrityl)-4-(hydroxymethyl)-N-((2,2,2-trifluoroacet-1-yl)-4,9,13-triazatridecane)-butyramide]phosphoramidite* (**17**). **17** was synthesized according to procedure described for **11**. (0.155 g, 55% yield). ^1^H-NMR (CDCl_3_): δ (ppm) 6.76–7.4 (m, 13H, DMT), 4.22 (dt, 1H, CO*CH*OH), 3.74 (s, 6H, 2OCH_3_), 3.12–3.55 (m, 16H, *CH_2_*OH, CONH*CH_2_* (spermine), CH_2_OP), 2.56–2.62 (m, 2H, 2CH, i-Pr), 2.24–2.42 (m, 4H, OCH*CH_2_*, CH_2_CN), 1.57–1.84 (m, 8H, spermine), 1.1–1.2 (m, 12H, 4CH_3_, i-Pr). ^31^P-NMR (C_6_H_6_): δ (ppm) 151.73; 151.59; 148.25; 147.83. MS (ESI) *m*/*z*: calcd for C_50_H_64_F_9_N_6_O_9_P [M + K]^+^ 1133.3966, found 1133.303; calcd for C_50_H_64_F_9_N_6_NaO_9_P [M + Na]^+^ 1117.4226, found 1117.45.

### 3.3. Oligonucleotide Preparation 

The DNA dodecamers (5′-CTC AAG CAA GCT-3′, 5′-AGC TTG CTT GAG-3′, 5′-CTC ACA TGC GCG-3′, 5′-CGC GCA TGT GAG-3′) were synthesized using DNA synthesizer Gene Assembler Plus from Pharmacia-LKB (Uppsala, Sweden) or K & A Laborgerate GbR DNA/RNA (Frankfurt am Main, Germany), using standard phosphoramidite chemistry. The non-nucleosidic analogues (**6**, **11**, **11a**, **17**) were inserted at positions marked with an X as listed in the Table 1. For the modified phosphoramidites, two-fold excess of phosphoramidites (in comparison to the standard protocol) and a prolonged coupling step of 10 min were used. The oligomers were cleaved from the CPG-support with 32% aqueous ammonia (room temperature, 1 h). The deprotection under standard conditions using concentrated aqueous ammonia at 55 °C overnight allowed for removal of all protecting groups, including trifluoroacetyls [3,38]. The oligomers were purified by the TLC on Merck 60 F_254_ TLC plates with *n*-propanol/aqueous ammonia/water solution (55:35:10, by vol.) as an eluent. The product band (least mobile) was cut out, eluted with water and desalted with Waters Sep-pak C-18 cartridges. First, the solution containing the oligonucleotide was loaded onto the cartridge and the column was flushed with 10 mM ammonium acetate (10 mL). In the next step, the oligonucleotides were eluted by flushing the cartridge with 5 mL of 30% acetonitrile/water solution. The fraction with the product was evaporated to dryness and the purity of oligonucleotides was monitored using HPLC and confirmed (by MALDI-TOF spectrometry Autoflex, Bruker). Oligonucleotides were also purified in a subsequent reverse phase HPLC step (UFLC system with LC-20AD pump; C(18)2 100 Å column (15 cm × 4.6 mm); starting from 0.01 M triethylammonium acetate (pH 7.0) up to CH_3_CN:CH_3_COOEt_3_N (40%/60%). Identity was confirmed by mass spectroscopy on MALDI-TOF (Autoflex, Bruker Daltonik GmbH, Bremen, Germany). The isolated yields of modified oligonucleotides were at the range of 23%–57%: 26%–57% and 23%–26% for **ON3**–**ON6** and **ON7**–**ON9** respectively.

### 3.4. Thermodynamic Analysis 

UV melting profiles of the DNA duplexes were obtained in a buffer containing 100 mM sodium chloride, 20 mM sodium cacodylate, 0.5 mM Na_2_EDTA, pH 7.0. Duplexes were used in the 10^−3^–10^−6^ M concentration range. Single strand concentrations were calculated from absorbance above 80 °C with single strand extinction coefficients approximated by the nearest-neighbor model [51]. The temperature range, in a heating-cooling cycle, was 0–90 °C with a temperature gradient of 1 °C/min. Thermal-induced transitions of each mixture were monitored at 260 nm with a Beckman DU 650 spectrophotometer with a temperature controller. The thermodynamic parameters were determined from fits of data acc. to a two-state model with the MeltWin 3.5 software [52].

## 4. Conclusions

Nucleic acids that carry polycationic modifications have many therapeutic and biotechnological advantages. Our studies corroborate that non-nucleosidic analogues of polyaminooligonucleosides maintain affinity and easily form duplexes with complementary strands. In some cases, this may increase the stability of modified complexes of nucleic acids. These new properties based on the rationale of charge masking do not change the scheme of the secondary structure of nucleic acids. Thus, they can ease transferring of nucleic acids past cellular barriers.

## Supplementary Materials

Supplementary materials can be accessed at: http://www.mdpi.com/1420-3049/20/07/12652/s1.
