# Supplementary material for: Non-Nucleosidic Analogues of Polyaminonucleosides and Their Influence on Thermodynamic Properties of Derived Oligonucleotides
Source: Molecules. 2015 Jul 13;20(7):12652–69. doi: 10.3390/molecules200712652 (PMC6332422; doi:10.3390/molecules200712652)
Supplement: Supplementary file 1 [file molecules-20-12652-s001.pdf]

Supplementary Materials

Content

NMR spectra **S1–S9**
MALDI-TOF spectra **S10–S12**
UV melting Job plots **S13–S15**

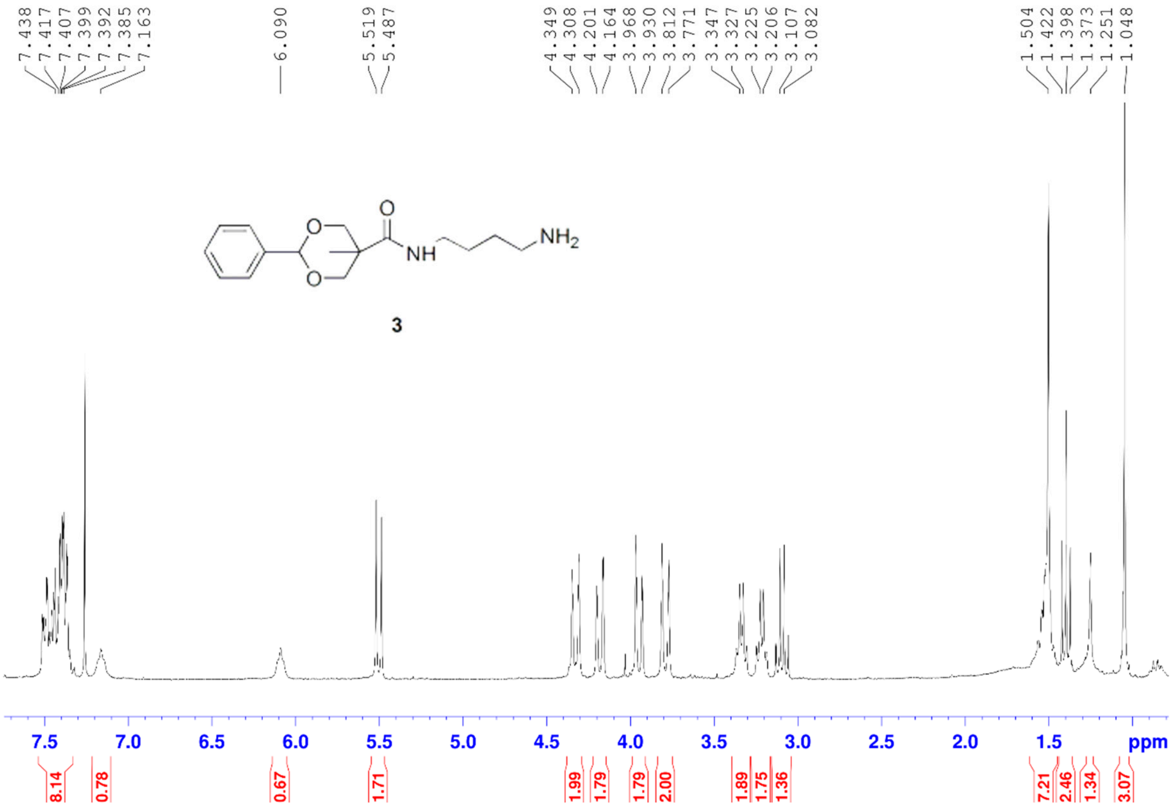

Figure S1. *N*-(4-Aminobutyl)-5-methyl-2-phenyl-1,3-dioxane-5-carboxamide.

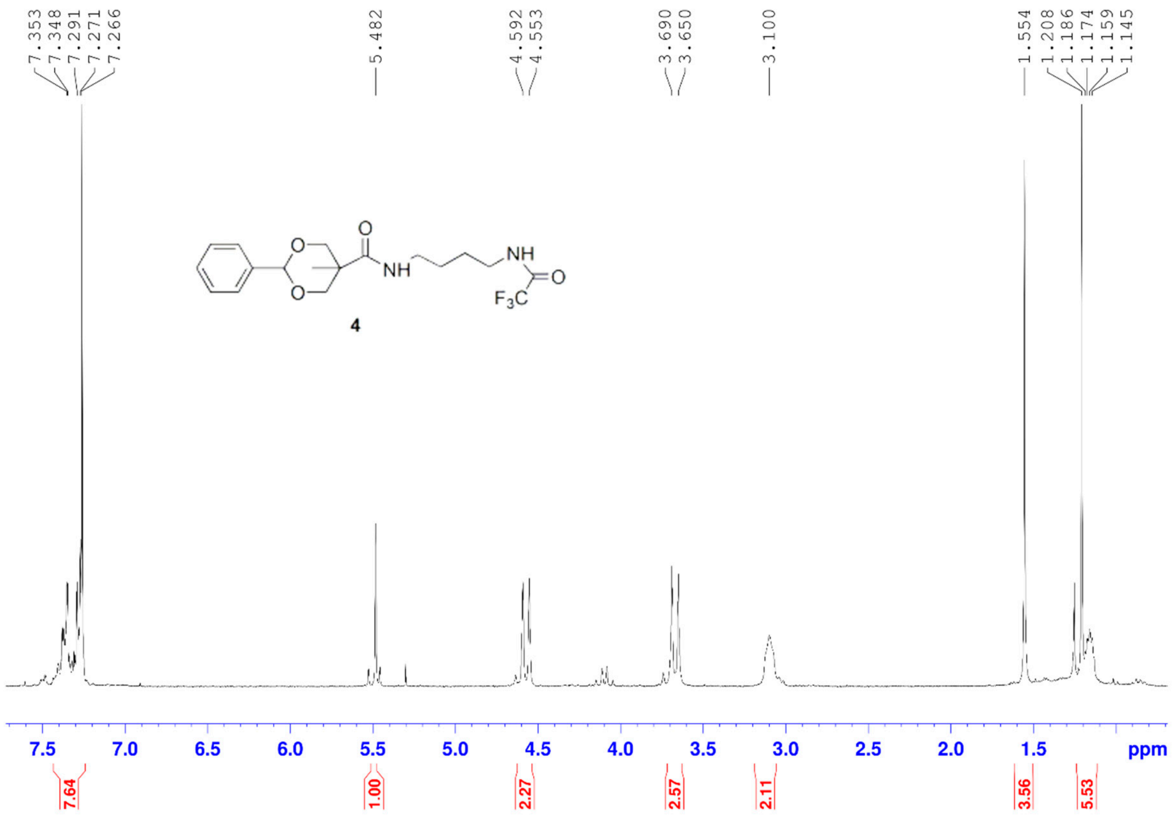

Figure S2. 5-Methyl-2-phenyl-*N*-(4-(2,2,2-trifluoroacetamid)butyl)-1,3-dioxane-5-carboxamide.

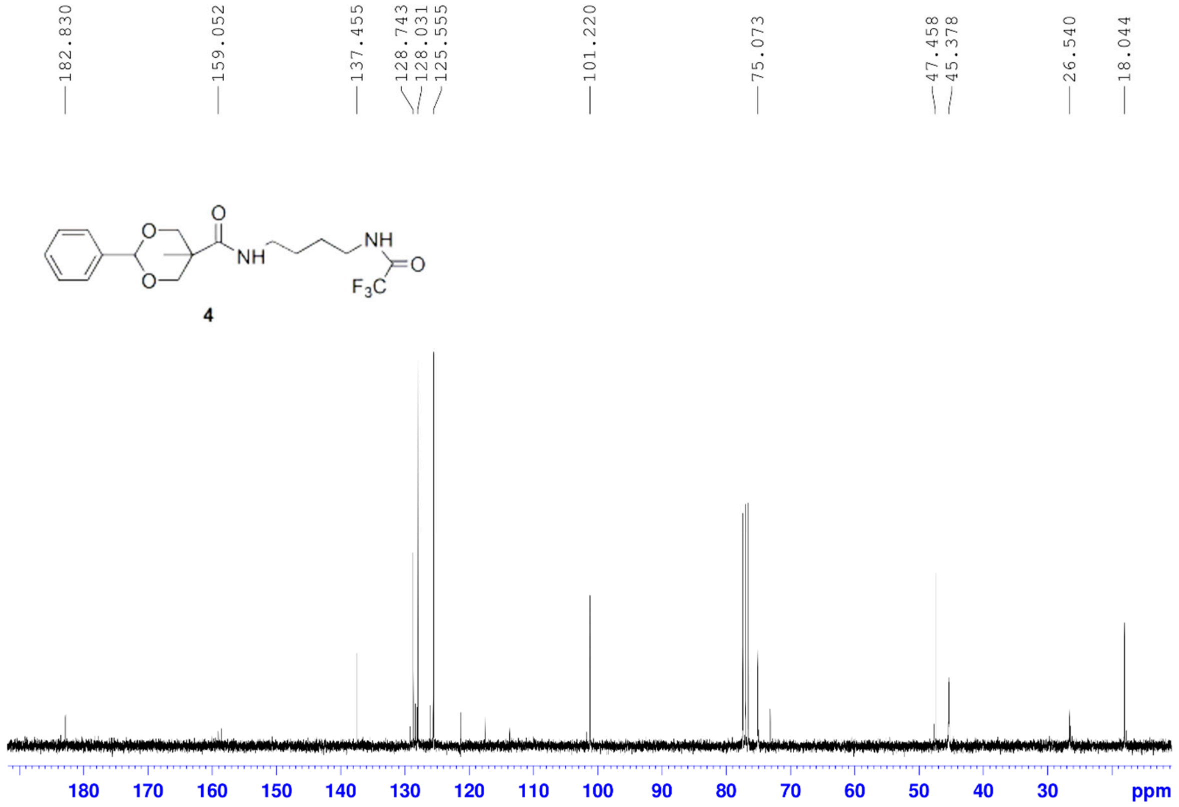

Figure S3. 5-Methyl-2-phenyl-*N*-(4-(2,2,2-trifluoroacetamid)butyl)-1,3-dioxane-5-carboxamide.

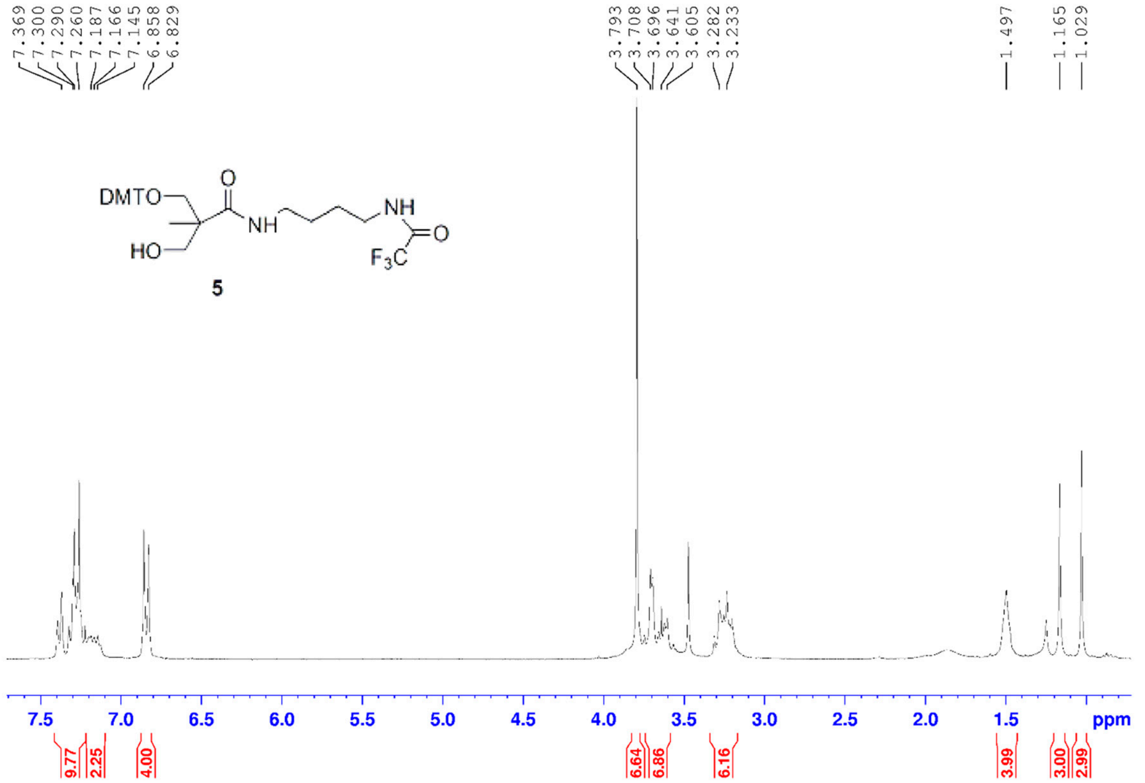

Figure S4. 3-(4,4′-Dimethoxytrityl)-2-(hydroxymethyl)-2-methyl-*N*-(4-(2,2,2-trifluoroacetamido)butyl)propanamide.

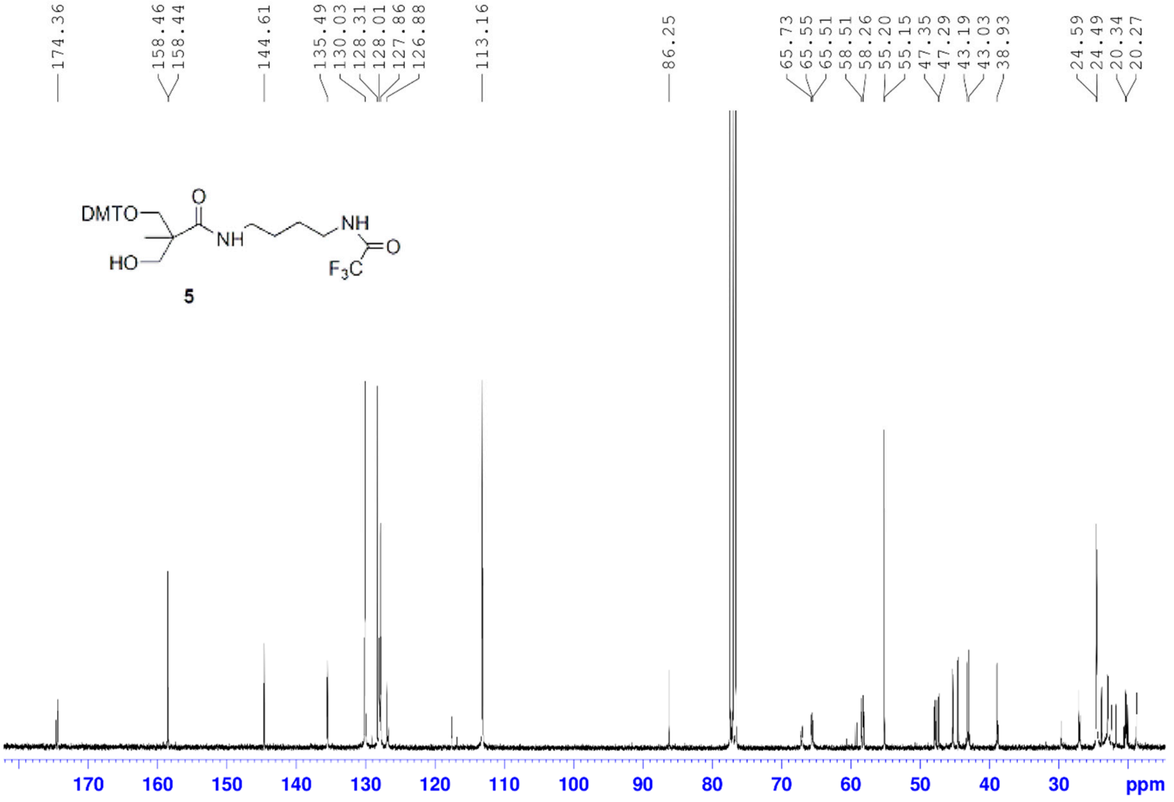

Figure S5. 3-(4,4′-Dimethoxytrityl)-2-(hydroxymethyl)-2-methyl-*N*-(4-(2,2,2-trifluoroacetamido)butyl)propanamide.

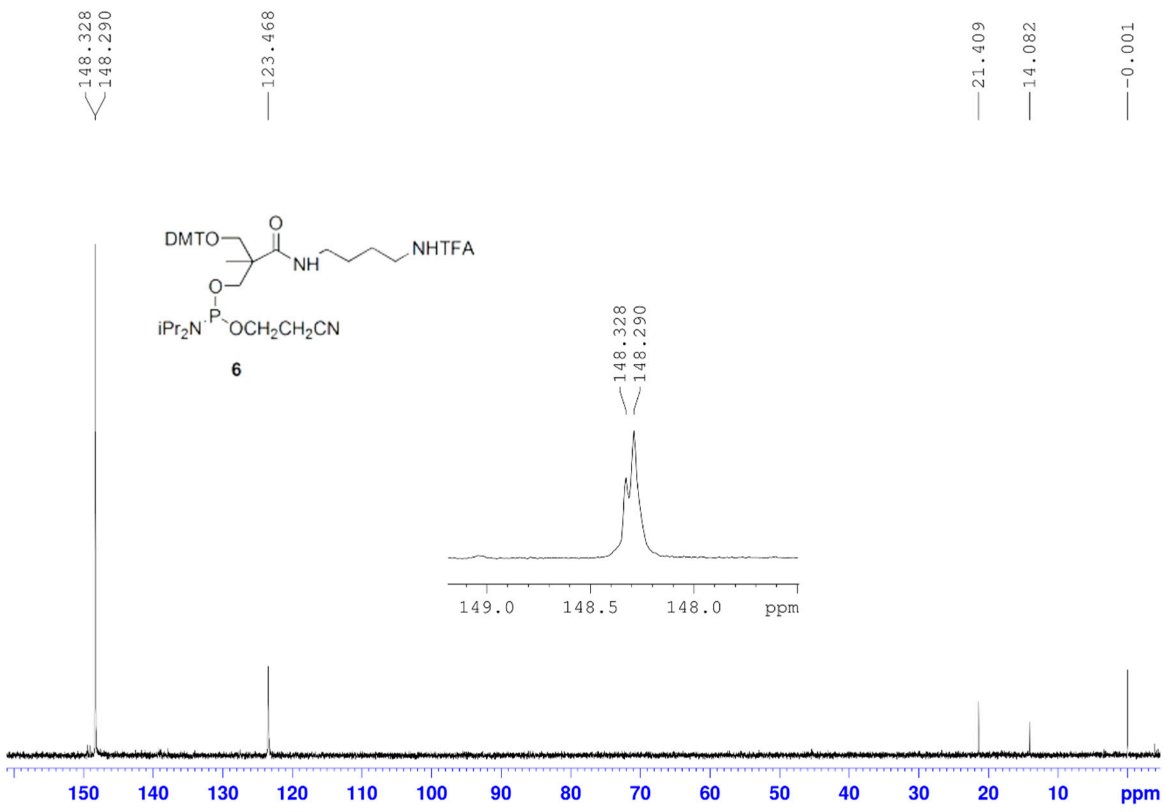

Figure S6. 3-[(4,4′-Dimethoxytrityl)-2-(hydroxymethyl)-2-methyl-*N*-(4-(2,2,2-trifluoroacetamido)butyl)propanamide]phosphoramidite.

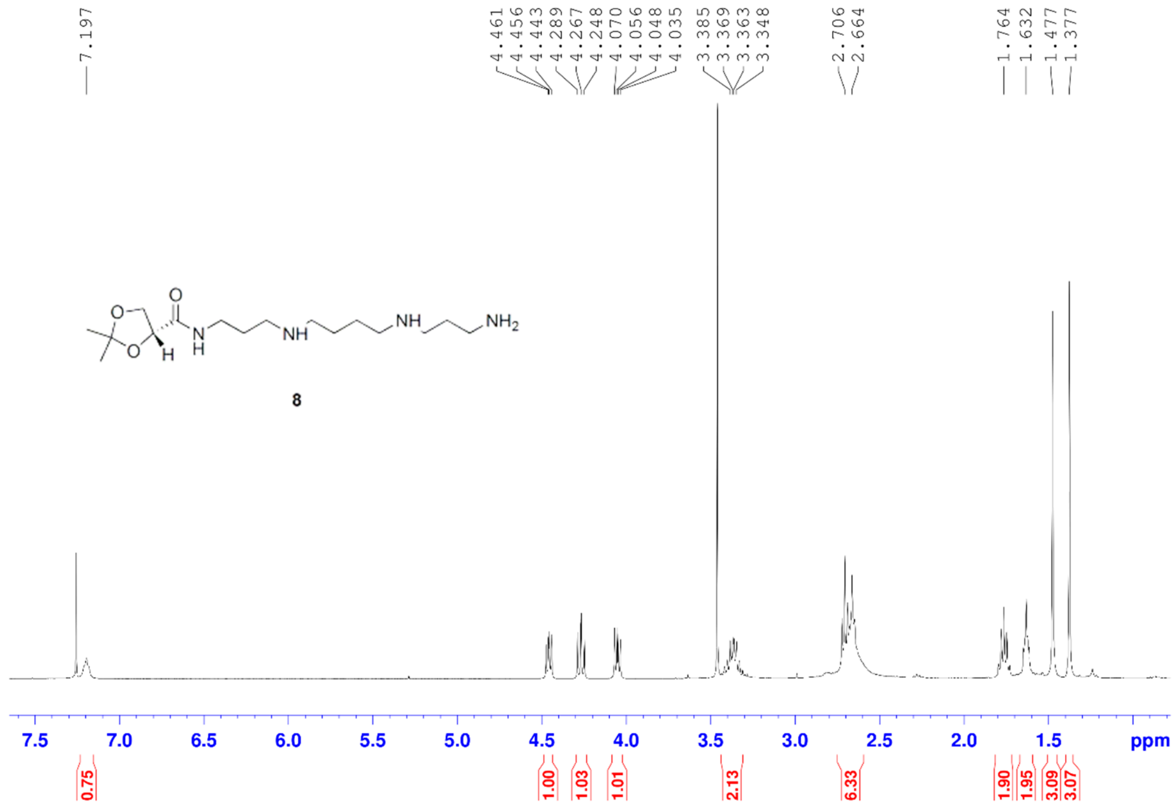

Figure S7. *(S)-N*-(4,9,13-triazatridecan-1-yl)-2,2-dimethyl-1,3-dioxolane-4-carboxyamide.

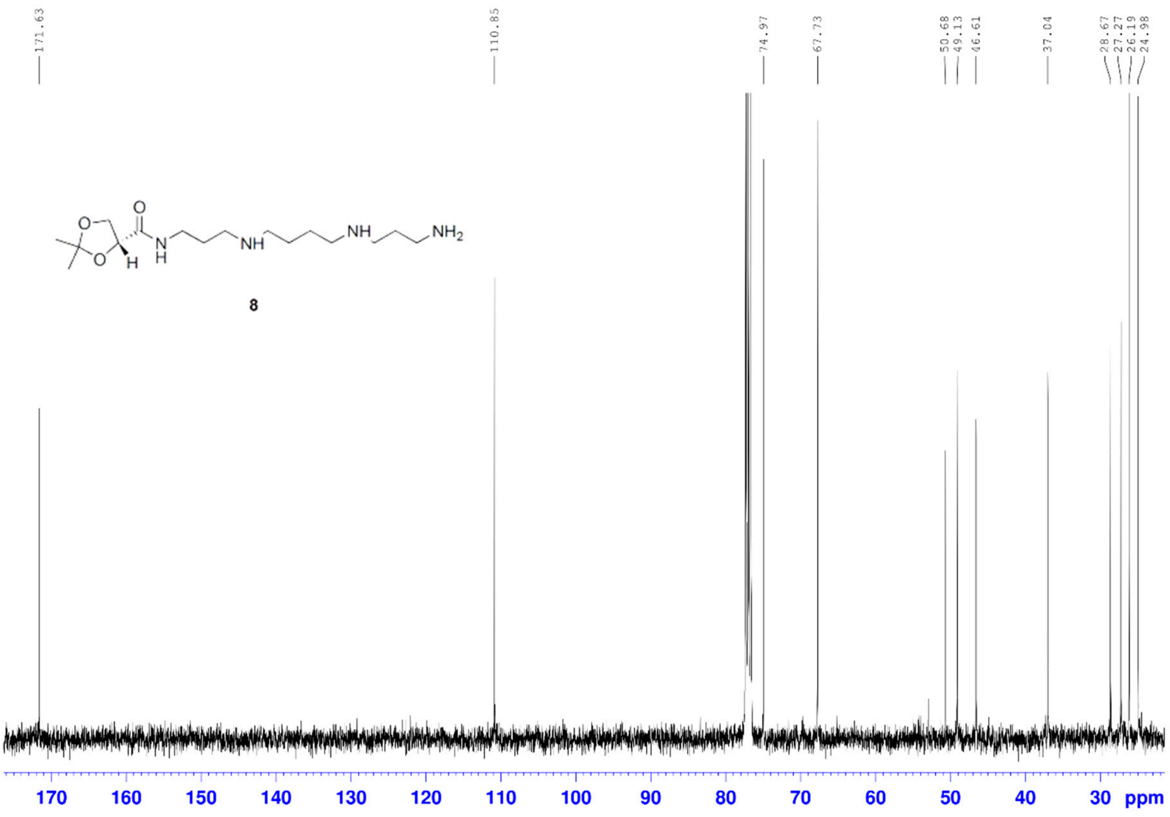

Figure S8. *(S)-N*-(4,9,13-triazatridecan-1-yl)-2,2-dimethyl-1,3-dioxolane-4-carboxyamide.

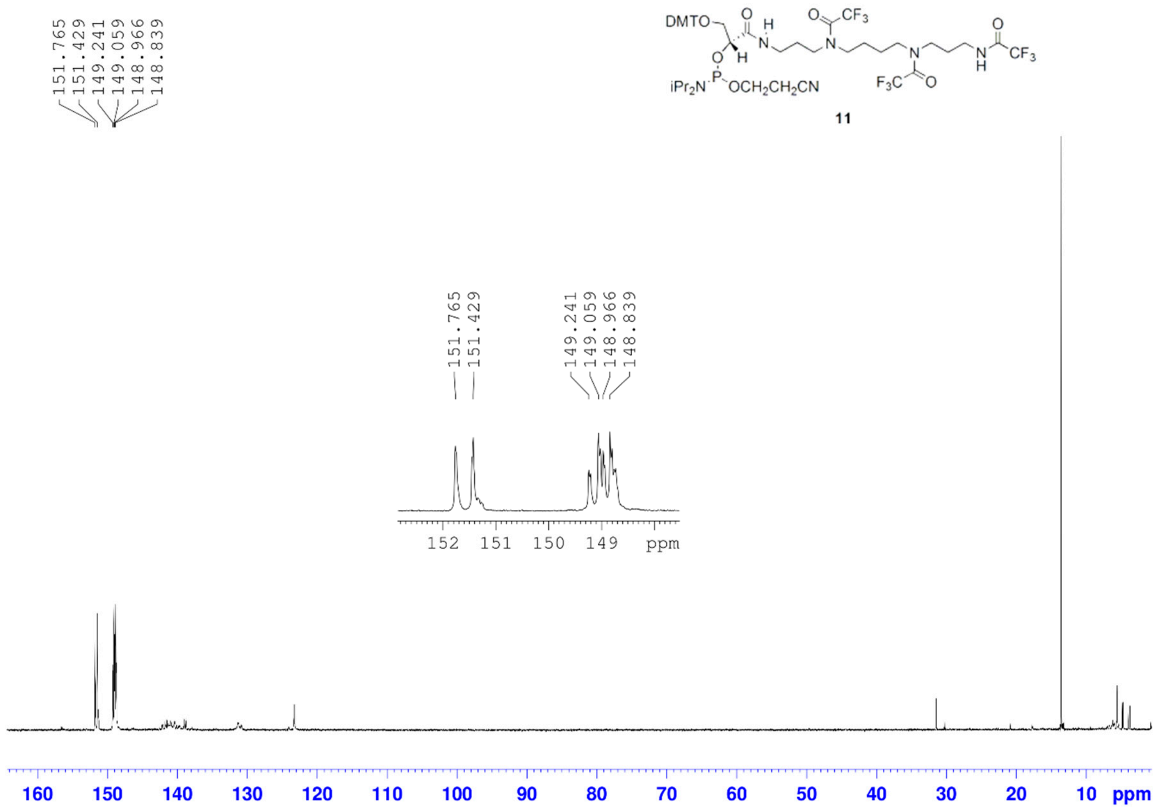

Figure S9. 2-*(S)*-[(4,4′-Dimethoxytrityl)-3-(hydroxymethyl)-*N*-((2,2,2-trifluoroacet-1-yl)-4,9,13-triazatridecane)propanamide]phosphoramidite.

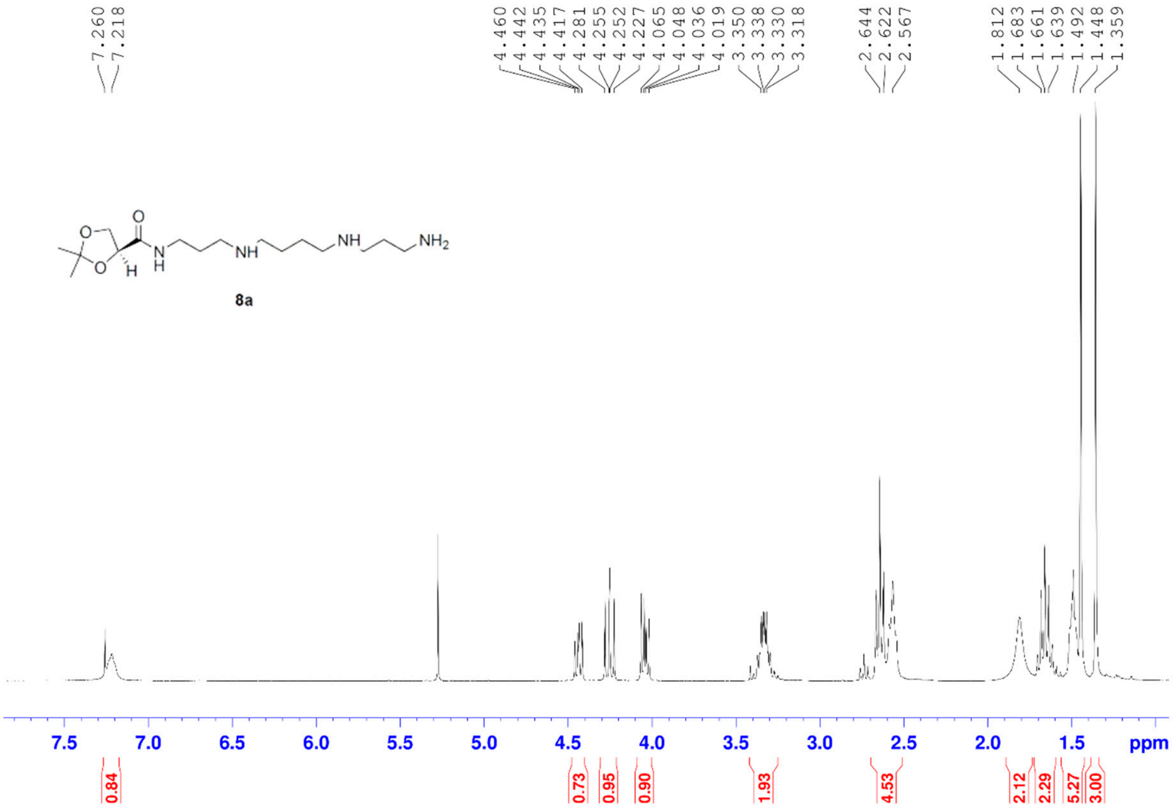

Figure S10. *(R)-N*-(4,9,13-triazatridecan-1-yl)-2,2-dimethyl-1,3-dioxolane-4-carboxyamide.

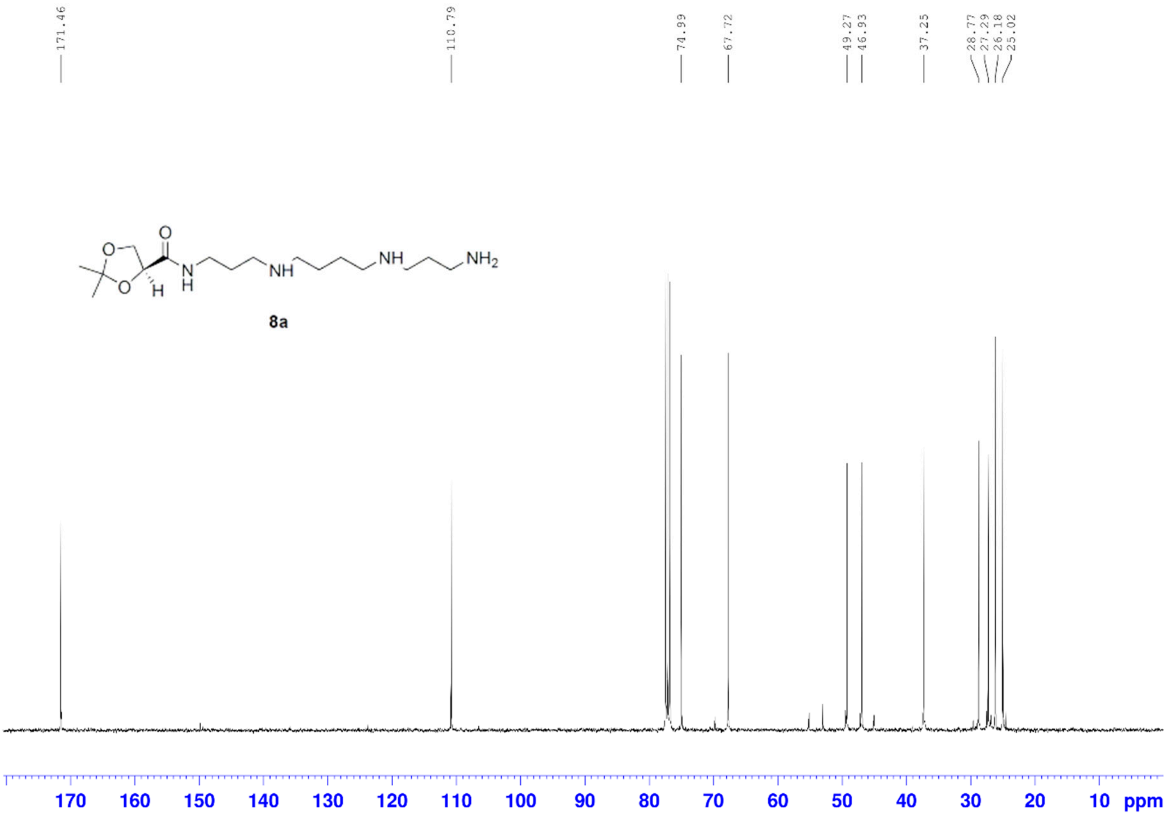

Figure S11. *(R)-N*-(4,9,13-triazatridecan-1-yl)-2,2-dimethyl-1,3-dioxolane-4-carboxyamide.

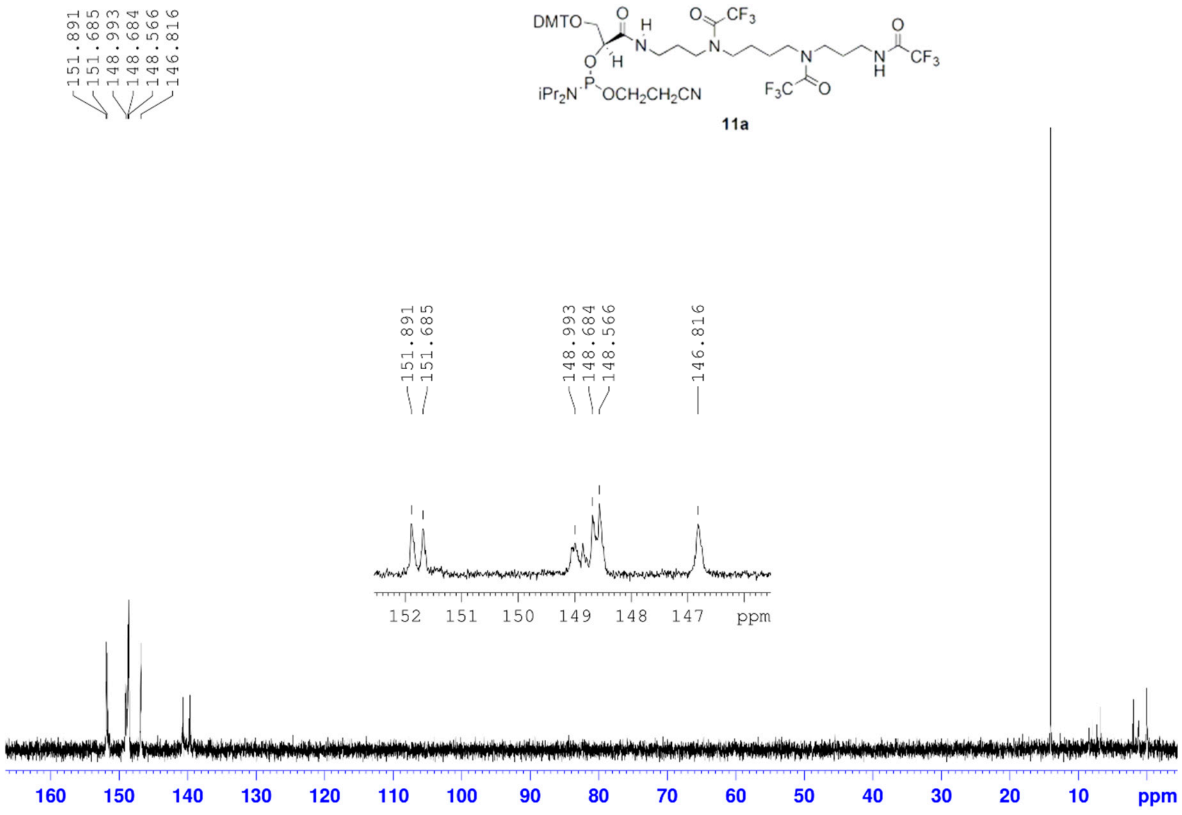

Figure S12. 2-*(R)*-[(4,4′-Dimethoxytrityl)-3-(hydroxymethyl)-*N*-((2,2,2-trifluoroacet-1-yl)-4,9,13-triazatridecane)propanamide]phosphoramidite.

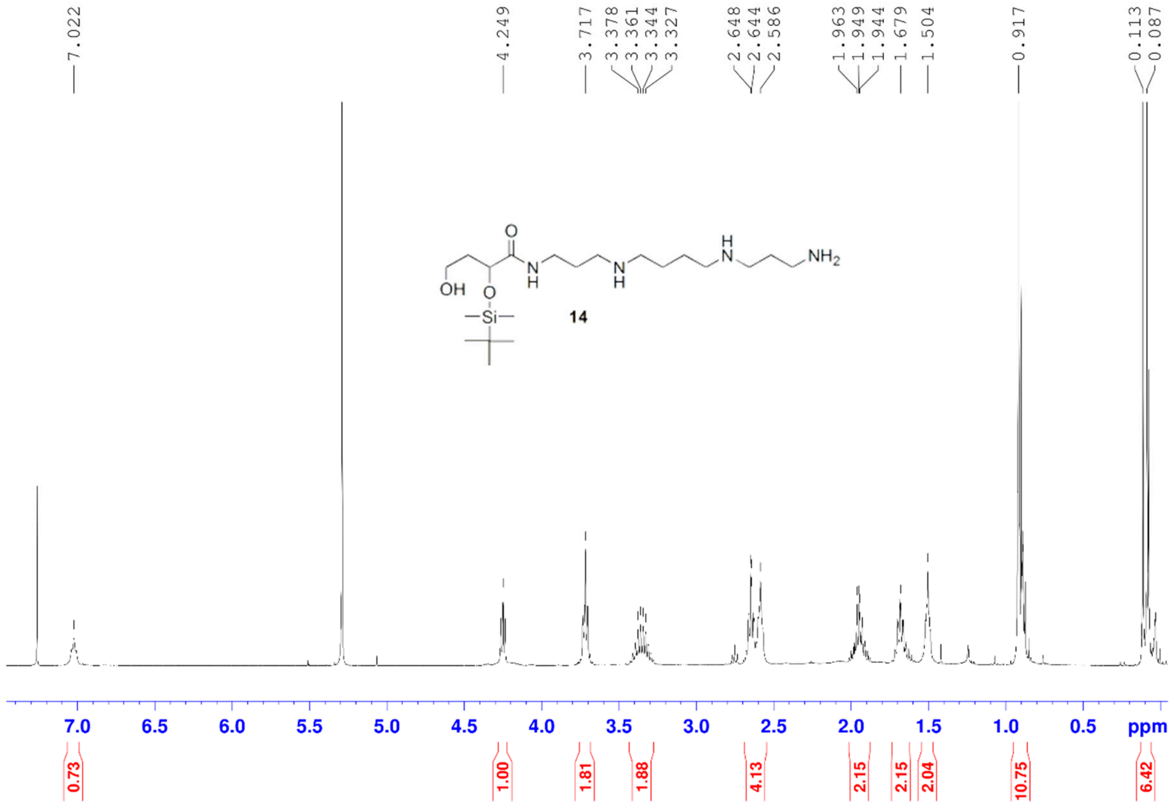

Figure S13. *N*-(4,9,13-Triazatridecan-1-yl)-2-(tert-butylodimethylsiloxy)-butyramide.

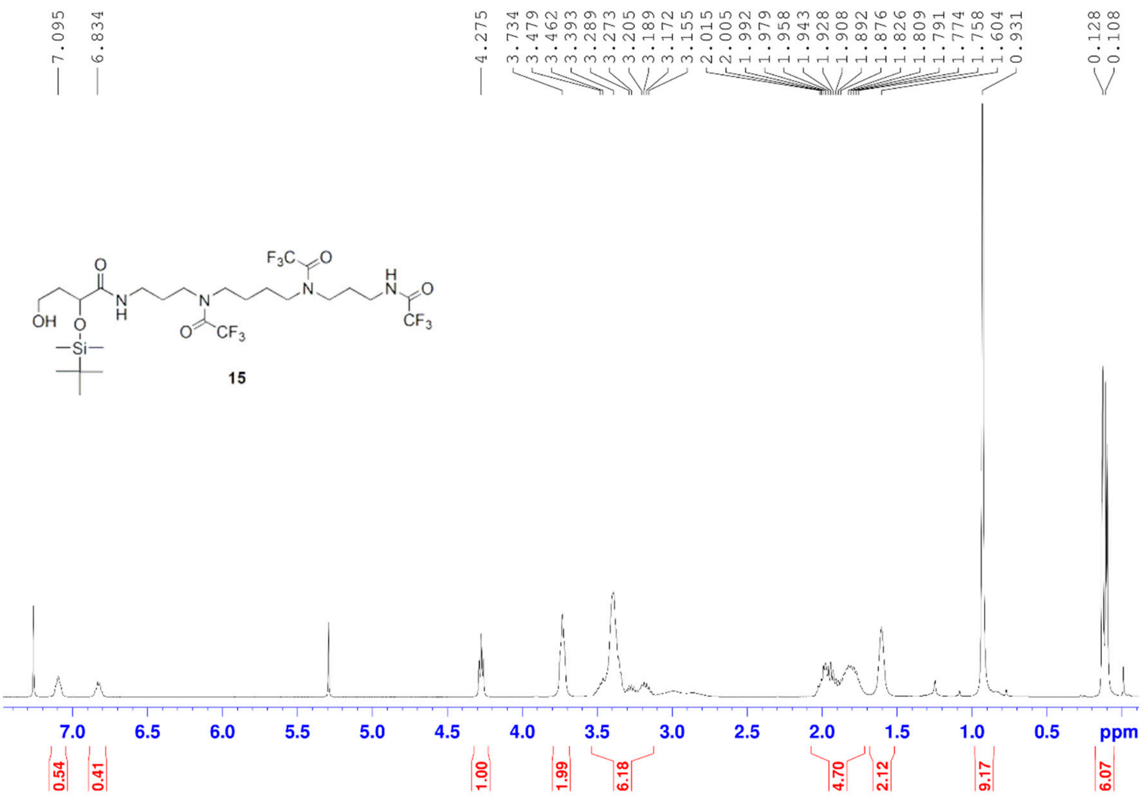

Figure S14. *N*-[Tris(2,2,2-trifluoroacet-1-yl)-4,9,13-triazatridecane]-2-(tert-butyldimethylsiloxy)-butyramide.

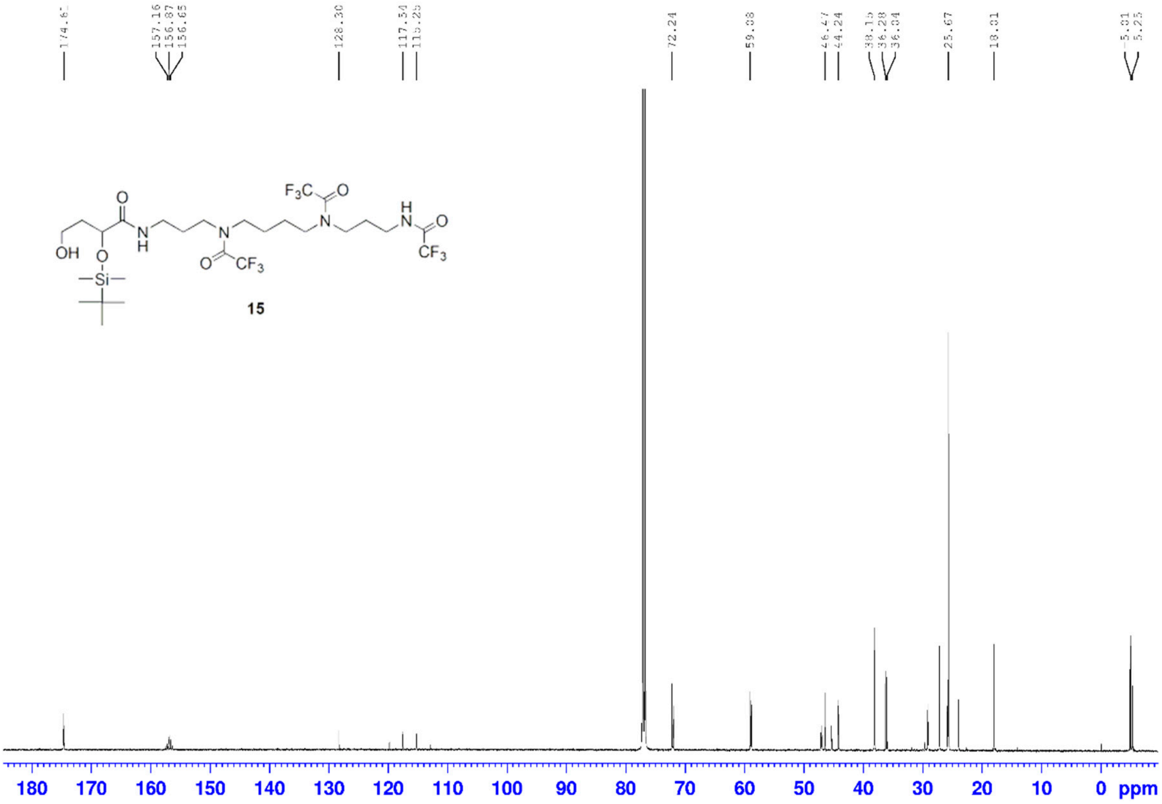

Figure S15. *N*-[Tris(2,2,2-trifluoroacet-1-yl)-4,9,13-triazatridecane]-2-(tert-butyldimethylsiloxy)-butyramide.

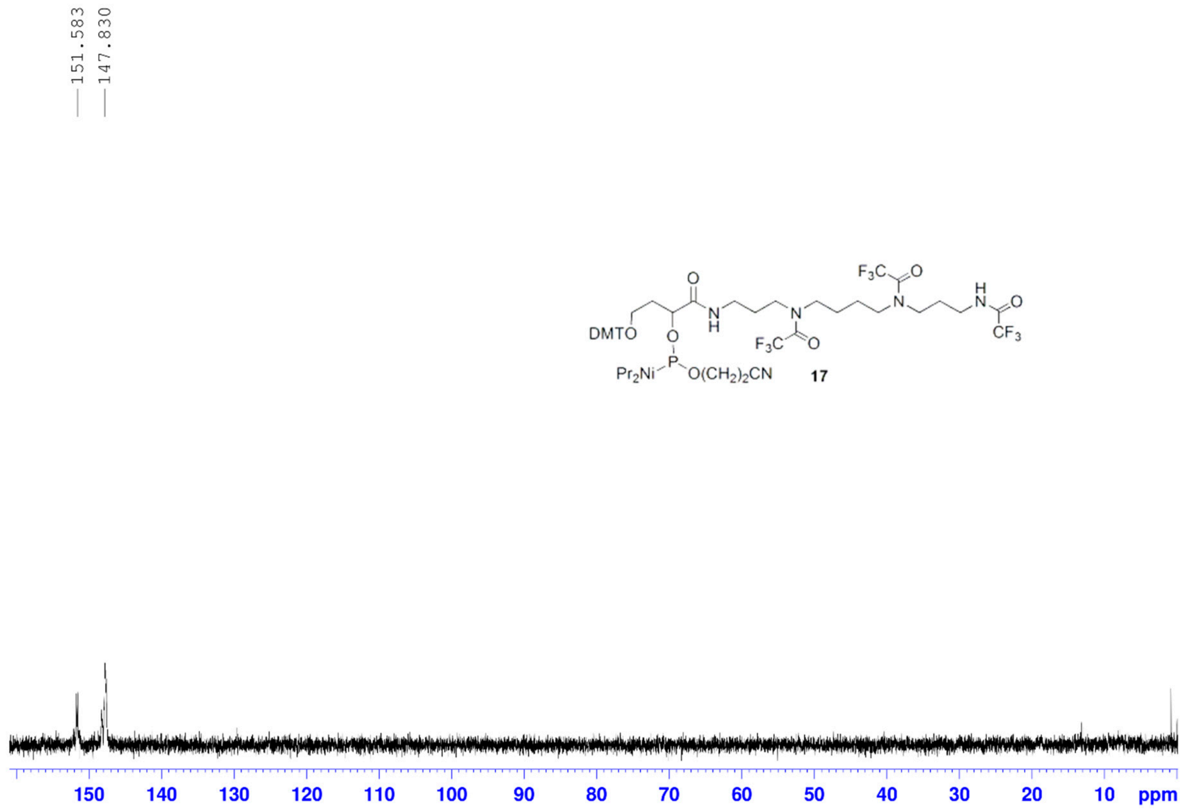

Figure S16. 2-(4,4′-Dimethoxytrityl)-4-(hydroxymethyl)-*N*-((2,2,2-trifluoroacet-1-yl)-4,9,13-triazatridecane)-butyramide]phosphoramidite.

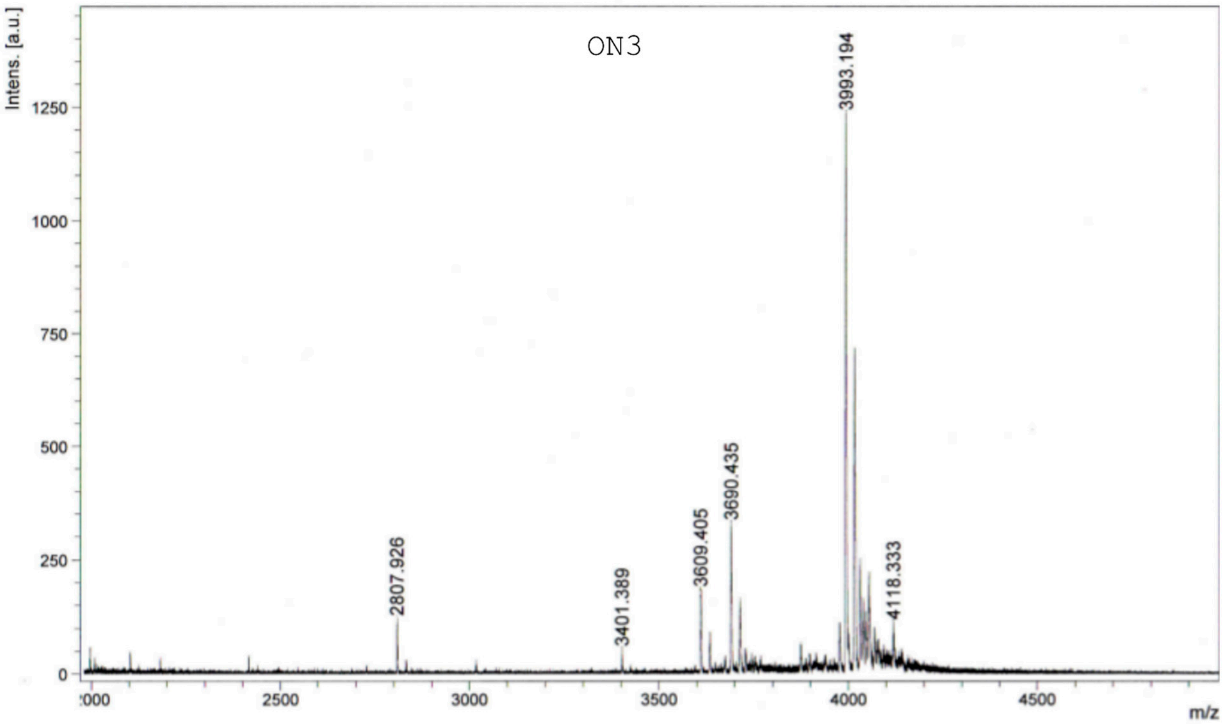

Figure S17. ON3 = X0 CTC AAG CAA GCT (X0 – putrescine analogue).

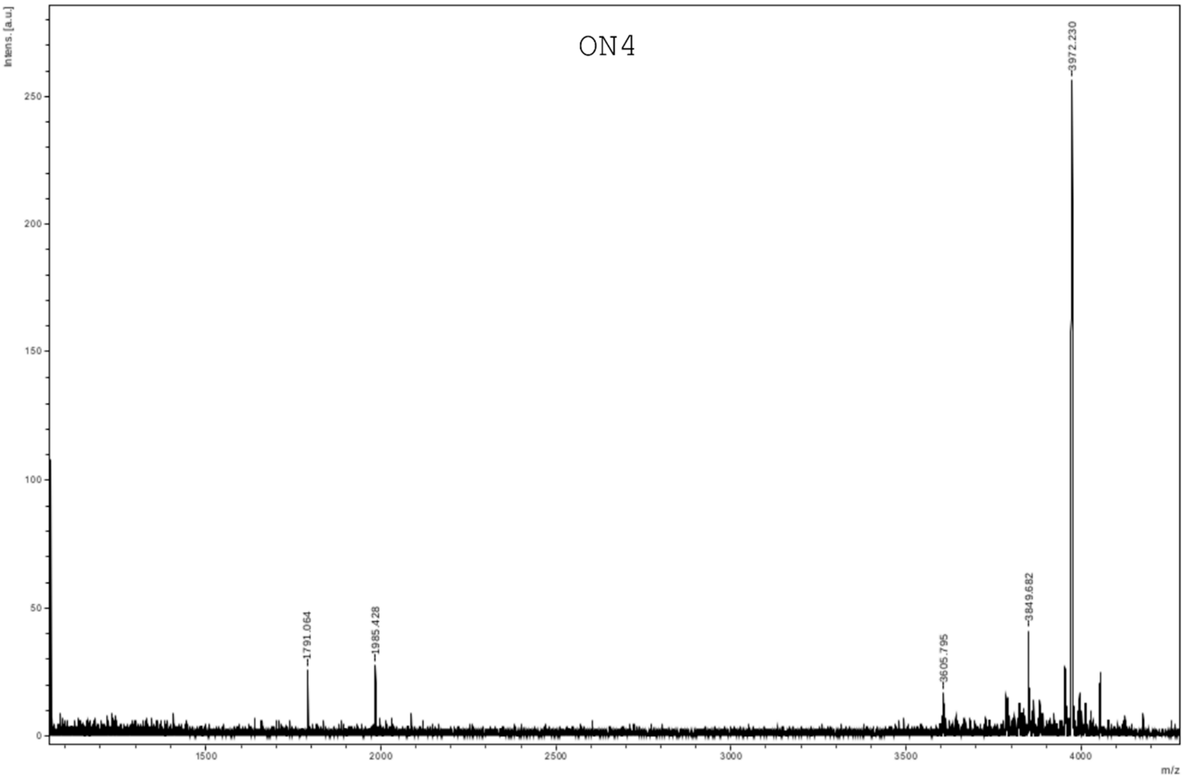

Figure S18. ON4 = X1 CTC ACA TGC GCG (X1 = spermine analogue).

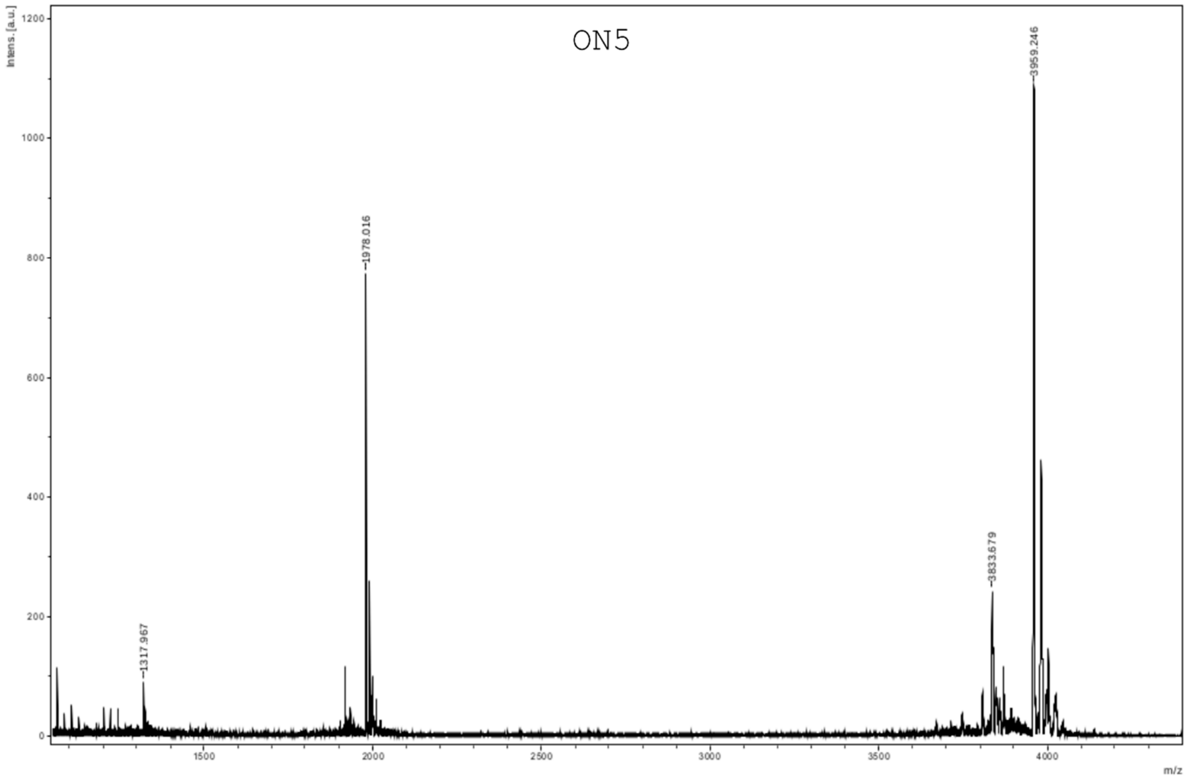

Figure S19. ON5 = X2 CTC ACA TGC GCG (X2 − spermine analogue).

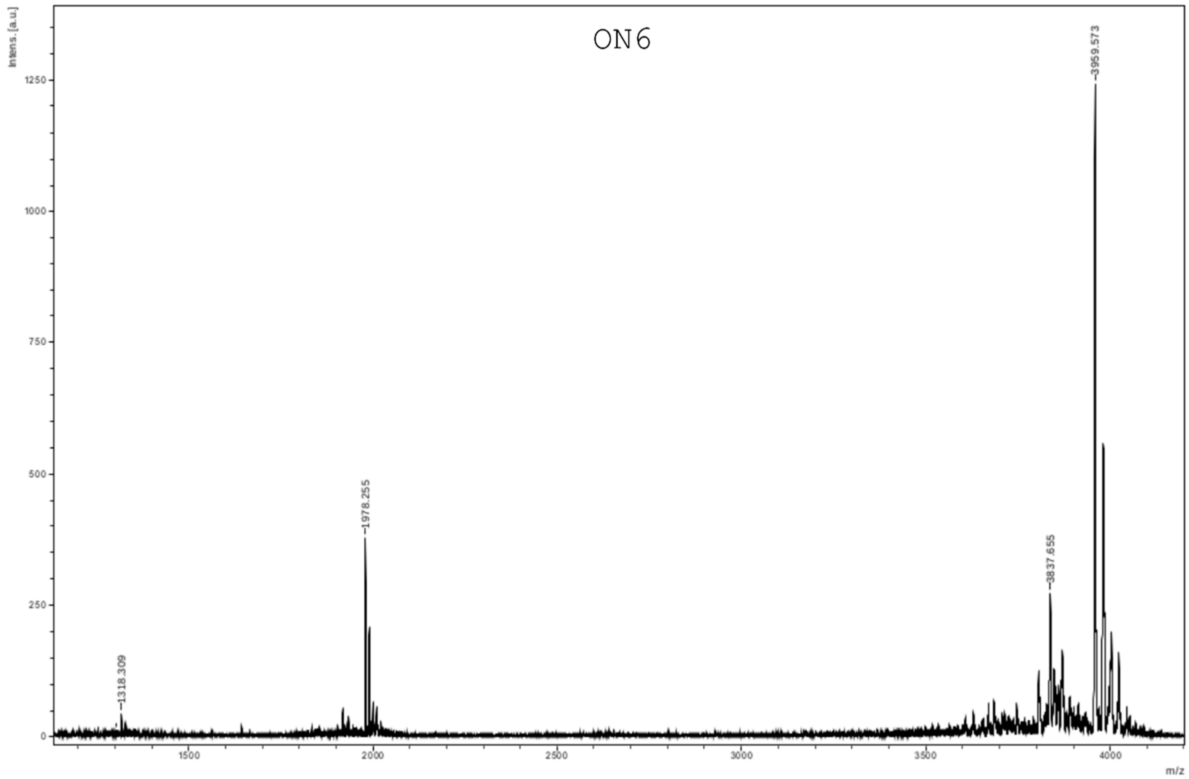

Figure S20. ON6 = X3 CTC ACA TGC GCG (X3 − spermine analogue).

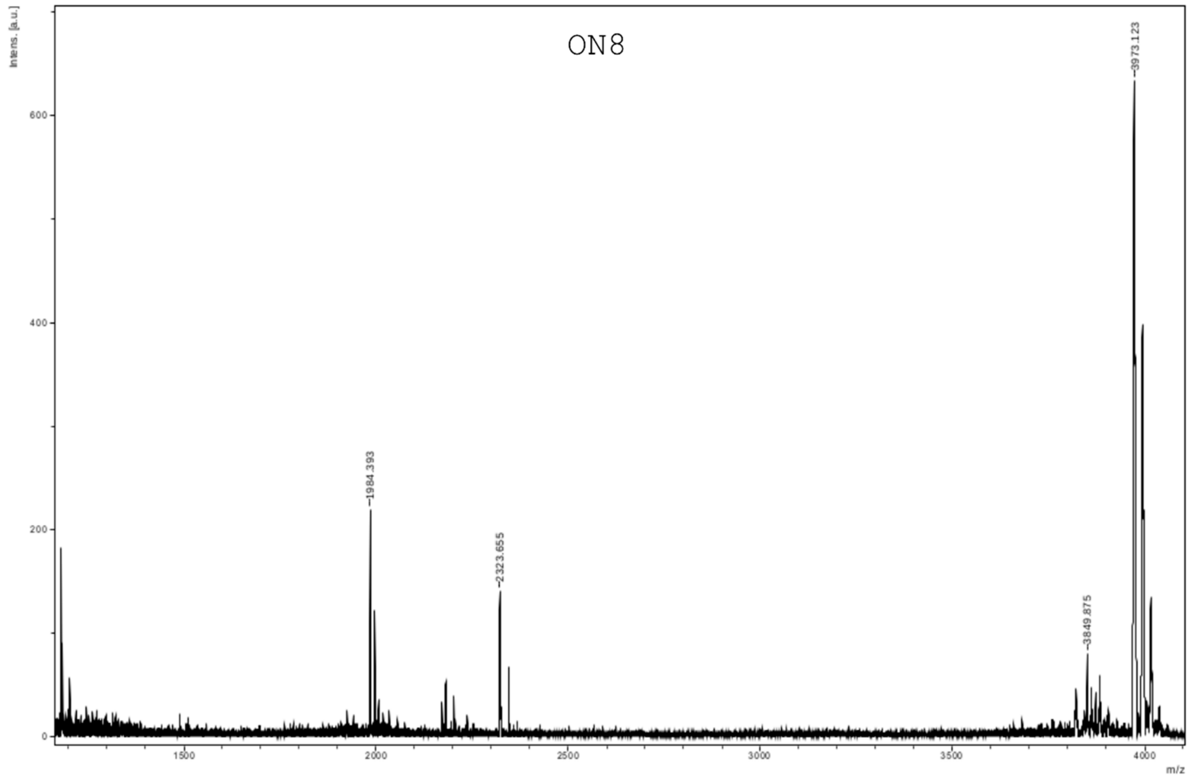

Figure S21. ON8 = CTC ACA X1 TGC GCG (X1 − spermine analogue).

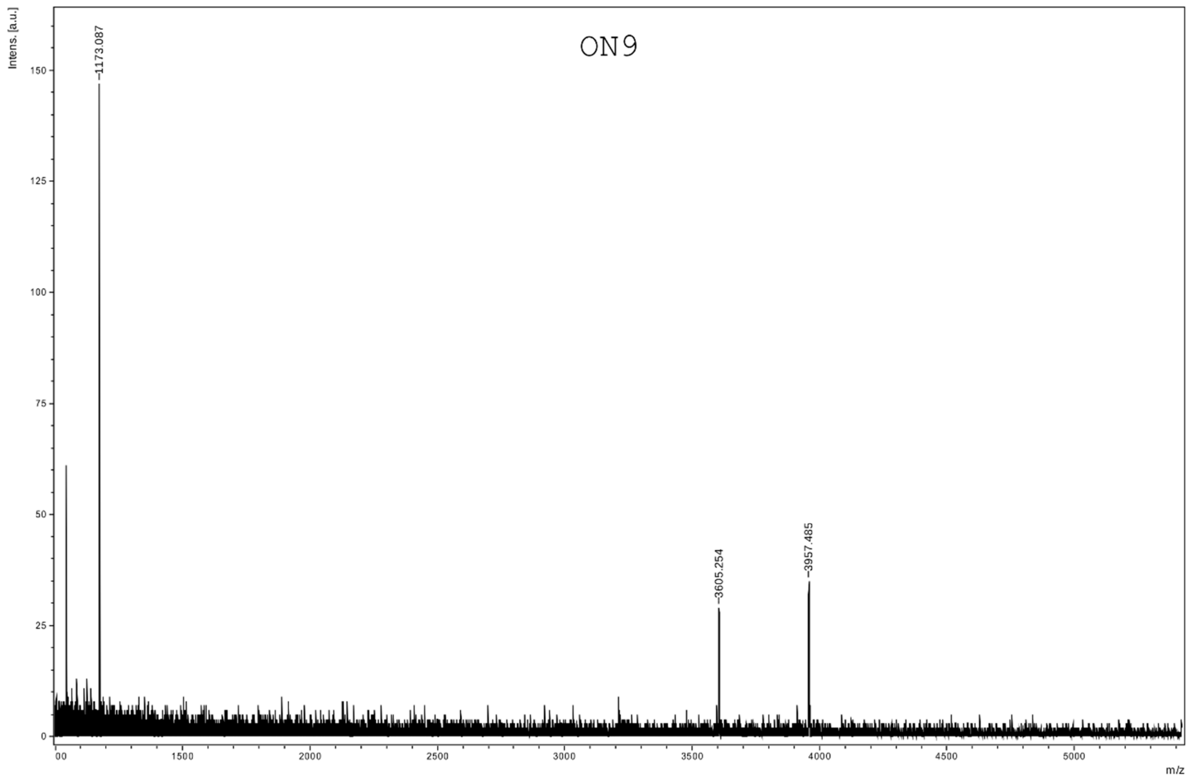

Figure S22. ON9 = CTC ACA X2 TGC GCG (X2 – spermine analogue).

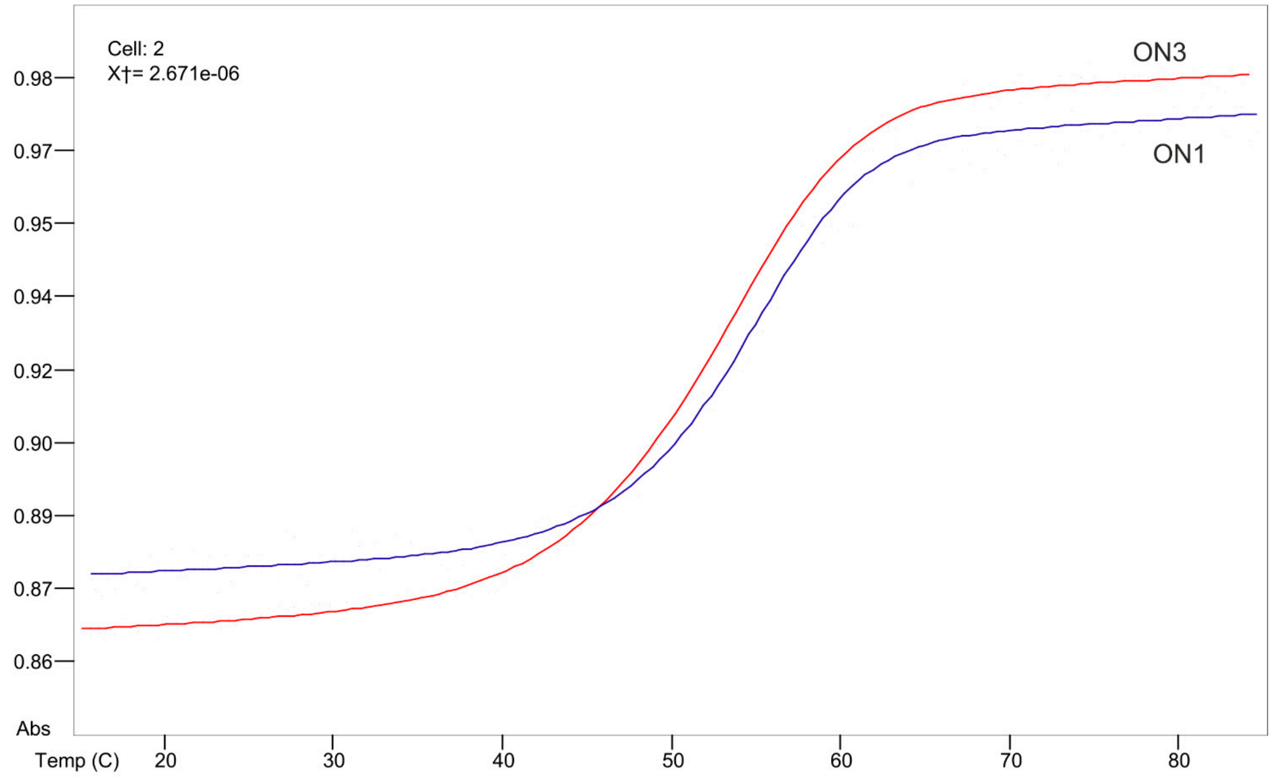

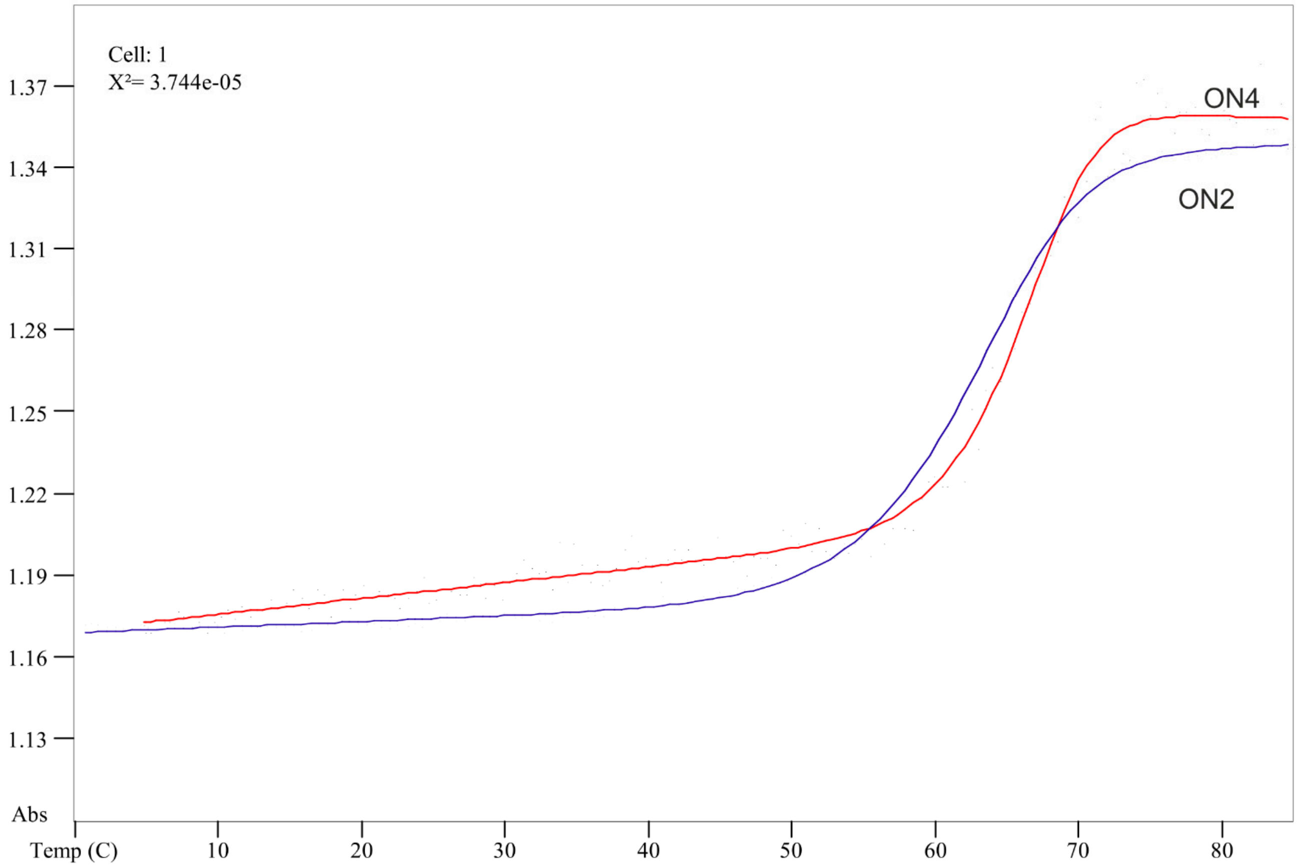

Figure S23. UV melting plots of comparison of thermodynamic stability for **ON1**/**ON3** and **ON2**/**ON4** (**ON1** and **ON2** − reference oligonucleotides).

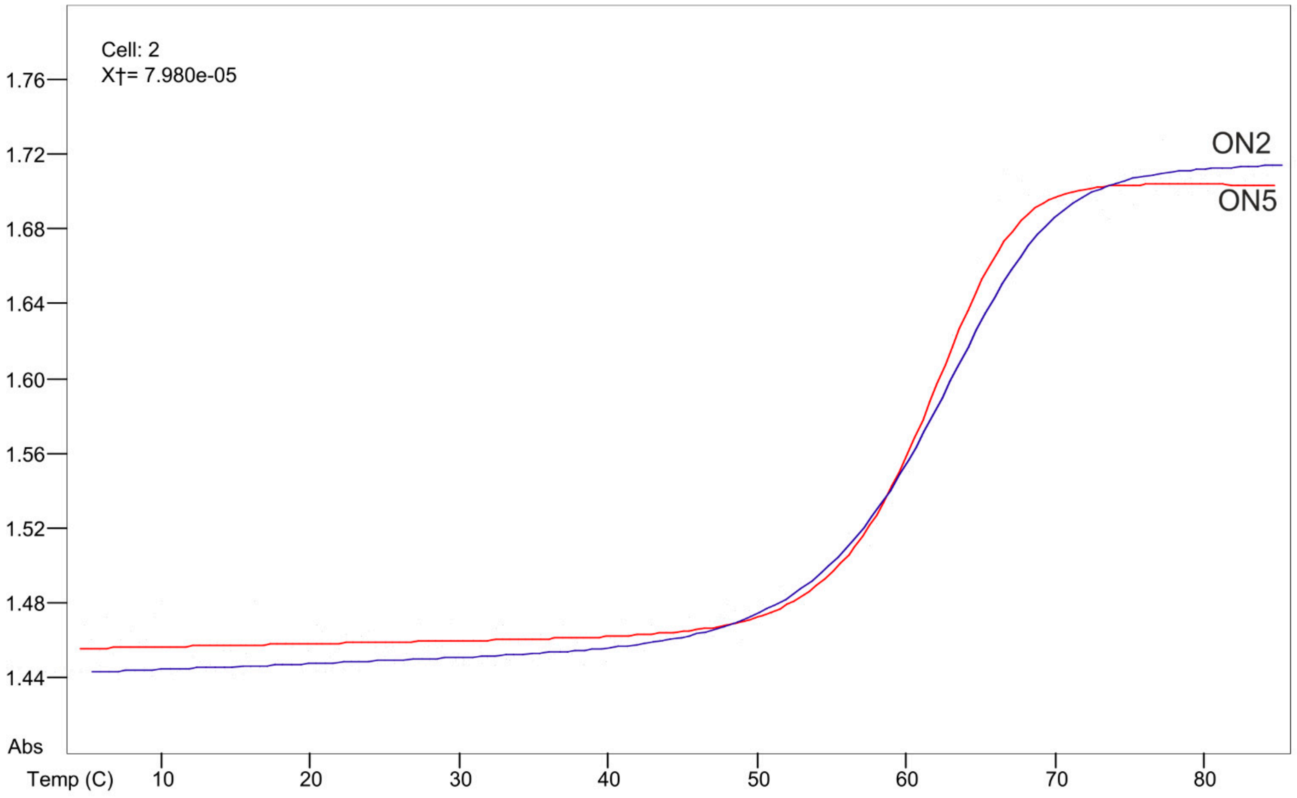

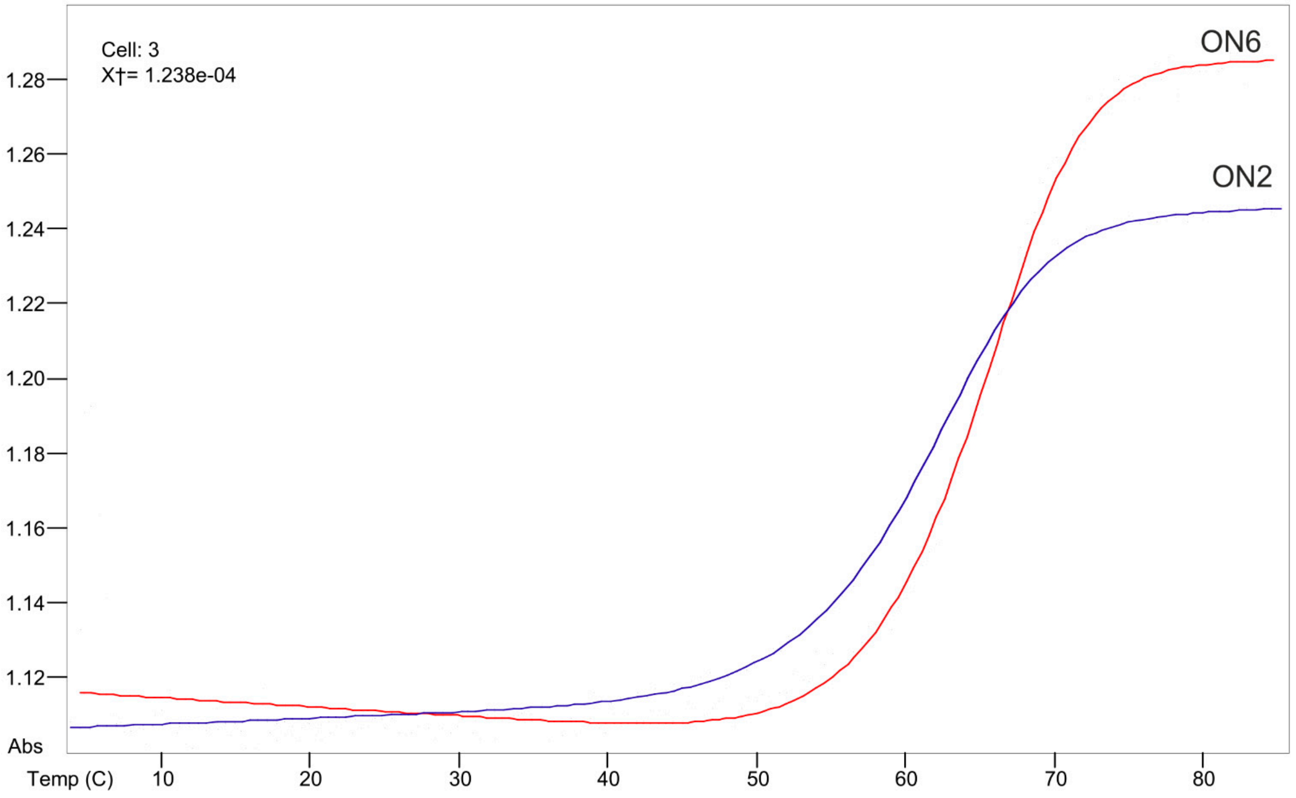

Figure S24. UV melting plots of comparison of thermodynamic stability for **ON2**/**ON5** and **ON2**/**ON6** (**ON2** − reference oligonucleotide).

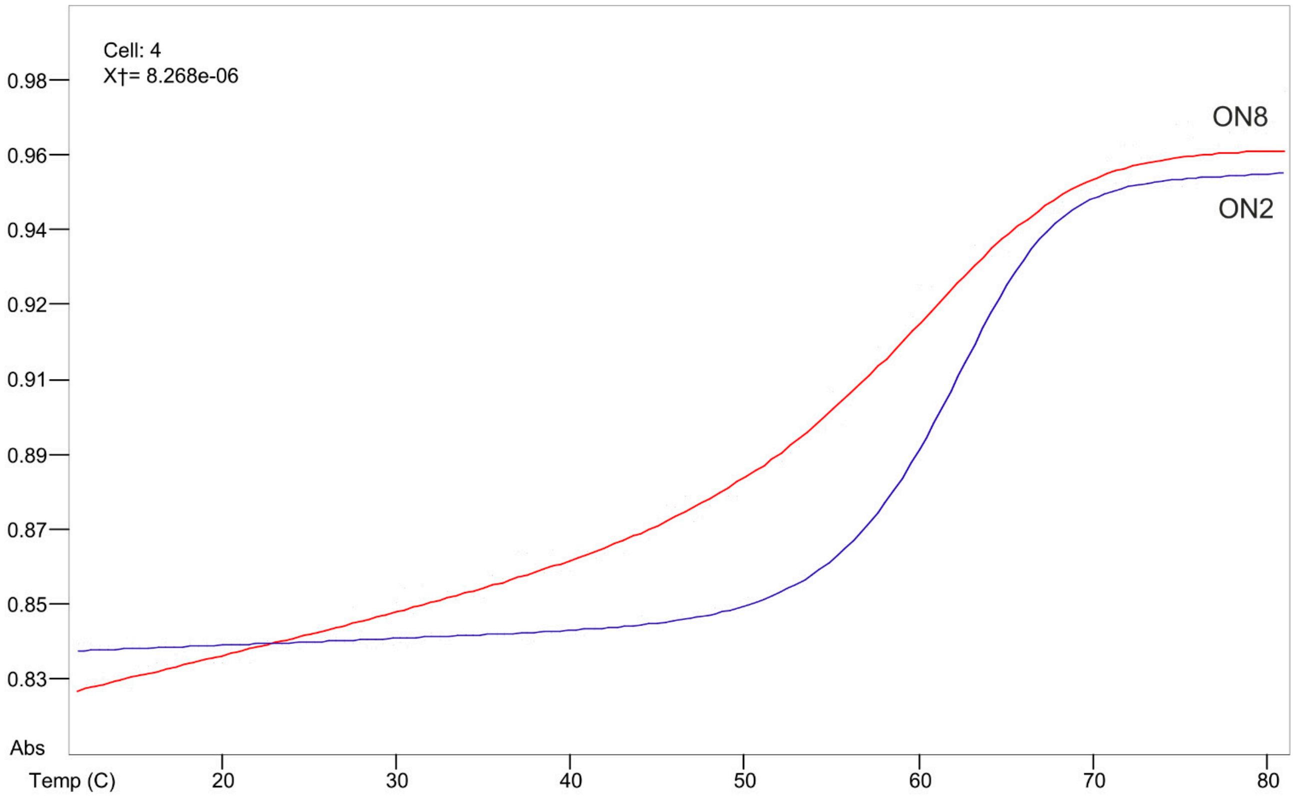

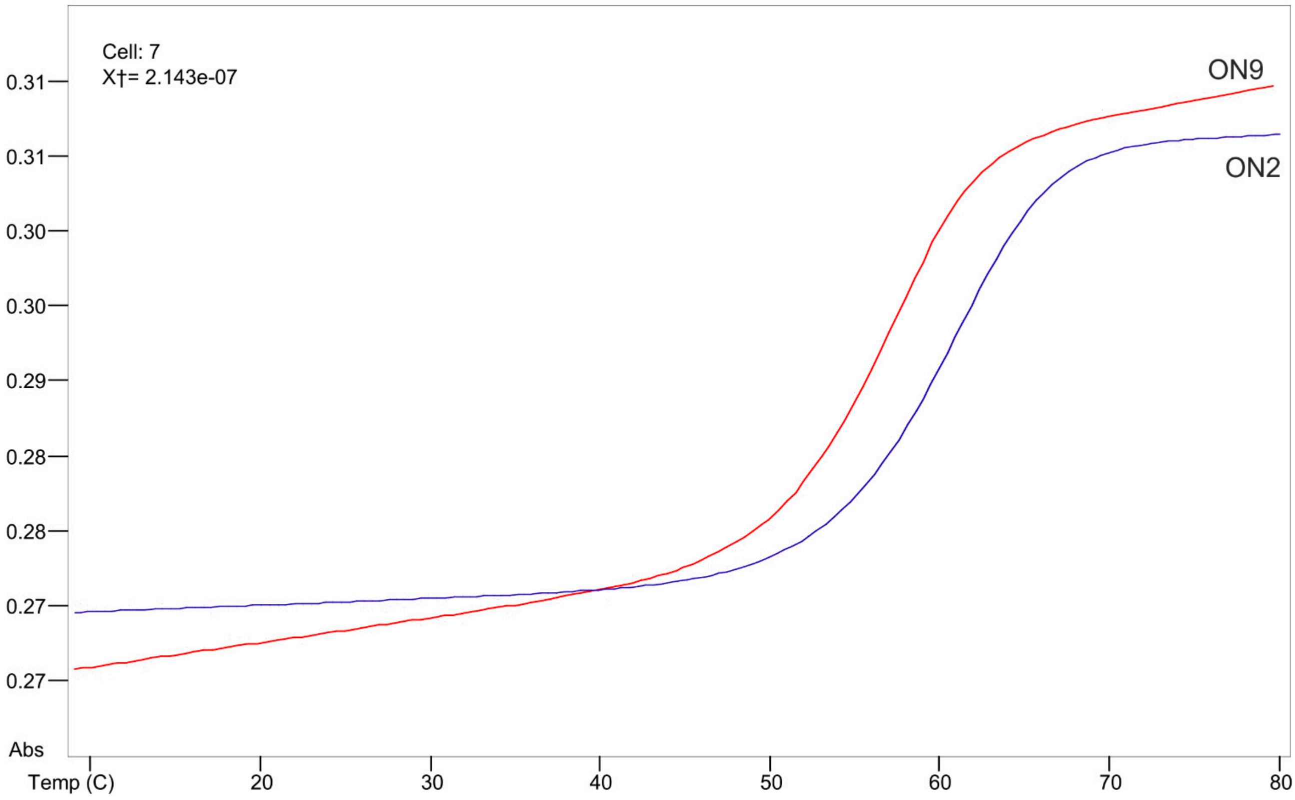

Figure S25. UV melting plots of comparison of thermodynamic stability for **ON2**/**ON8** and **ON2**/**ON9** (**ON2** − reference oligonucleotide).
